# Base Preferences in Non-Templated Nucleotide Incorporation by MMLV-Derived Reverse Transcriptases

**DOI:** 10.1371/journal.pone.0085270

**Published:** 2013-12-31

**Authors:** Pawel Zajac, Saiful Islam, Hannah Hochgerner, Peter Lönnerberg, Sten Linnarsson

**Affiliations:** Laboratory for Molecular Neurobiology, Department of Medical Biochemistry and Biophysics, Karolinska Institutet, Stockholm, Sweden; Centro de Biología Molecular Severo Ochoa (CSIC-UAM), Spain

## Abstract

Reverse transcriptases derived from Moloney Murine Leukemia Virus (MMLV) have an intrinsic terminal transferase activity, which causes the addition of a few non-templated nucleotides at the 3´ end of cDNA, with a preference for cytosine. This mechanism can be exploited to make the reverse transcriptase switch template from the RNA molecule to a secondary oligonucleotide during first-strand cDNA synthesis, and thereby to introduce arbitrary barcode or adaptor sequences in the cDNA. Because the mechanism is relatively efficient and occurs in a single reaction, it has recently found use in several protocols for single-cell RNA sequencing. However, the base preference of the terminal transferase activity is not known in detail, which may lead to inefficiencies in template switching when starting from tiny amounts of mRNA. Here, we used fully degenerate oligos to determine the exact base preference at the template switching site up to a distance of ten nucleotides. We found a strong preference for guanosine at the first non-templated nucleotide, with a greatly reduced bias at progressively more distant positions. Based on this result, and a number of careful optimizations, we report conditions for efficient template switching for cDNA amplification from single cells.

## Introduction

The introduction of second-generation massively parallel sequencing has revolutionized many areas in biological and medical research. One area greatly benefiting from the sequencing revolution is transcriptomics, where the massive output provided by modern sequencers has shed new light on the complexity of the RNA landscape. RNA studies using massively parallel sequencing are performed using a group of methods collectively termed RNA sequencing, or simply RNA-seq[1,2]. Briefly, in RNA-seq the sample of interest is isolated, the RNA is extracted and converted via a number of enzymatic steps into a sequencing library, i.e. to a format suitable for sequencing. Generally, this includes the introduction of appropriate adaptors to the ends of the molecules and size selection to arrive at homogenous fragment sizes from transcripts of different lengths. The library is thereafter sequenced, the obtained reads aligned to the genome and transcriptome with further analysis outlining the transcriptional landscape. The benefits of massive sequencing, as compared to the traditionally used microarrays, include a broad dynamic range, high accuracy and that no *a priori* information about the RNA is necessary.

Several different polymerases show nontemplated nucleotide addition at the 3´ end of an extended template[3]. For example, HIV reverse transcriptase[4] and Taq DNA polymerase both preferentially incorporate adenosines, whereas reverse transcriptases from the Moloney murine leukemia virus (MMLV), preferentially introduce cytosines[5]. 

Template switching is a mechanism by which reverse transcriptases (RTs) of the Moloney murine leukemia virus (MMLV) family can switch template from the RNA molecule to a secondary oligonucleotide, called the template-switching oligonucleotide (TSO), during cDNA synthesis. The template switch is enabled by the terminal transferase activity of the MMLV RTs that adds a couple of nucleotides in a template-independent fashion upon reaching the terminus of the RNA molecule. The TSO can transiently anneal to these protruding bases by virtue of a complementary ribonucleotide stretch. The RT then switches template from the RNA to the TSO and continues with the cDNA synthesis. In this manner, arbitrary sequences—for instance amplification handles or adapters—can be incorporated at both ends of the final cDNA molecule: at the 3´ end by tailing the oligo(dT) primer and at the 5´ end by designing a suitable TSO. For this reason, template switching is indeed employed in a number of commonly used protocols[6] [7]. 

In a number of instances, including development and cancer, studies at the single-cell level can provide information that is lost when analyzing whole populations of cells. Population-level studies generate average data over the entire cell pool that can mask contributions of important individual cells or cell types. It is therefore not surprising that the last couple of years, largely due to technological advances, have seen a great increase in the number of single-cell studies. For example, genomes of single cancer cells from a breast cancer have been sequenced to investigate the evolution and progress of this disease [8]. Furthermore, gene expression analysis of single circulating melanoma cells was used to identify potential biomarkers [9]. 

Single-cell studies are challenging because of the scarcity of the starting material. Preferably, the protocols should be simple with minimal numbers of enzymatic steps and purifications. Because of the straightforwardness and ease of implementation, template switching has found use in single-cell RNA seq library preparation. For example, a slightly modified version of Clontech’s SMARTer Ultra Low RNA Kit was recently combined with the standard Illumina library preparation procedure, as well as with the transposon-based Nextera library construction technique, in SMART-Seq – an RNA-seq protocol capable of transcriptome profiling of single cells [9]. 

Similarly, we have reported Single-cell tagged reverse transcription (STRT), an RNA-seq approach using template switching to introduce barcode and amplification sequences [10,11]. With STRT up to 96 single cells can be profiled in parallel, translating into reduced cost and increased throughput. Briefly, after cell isolation and lysis, each cell’s mRNA is converted to cDNA, which is simultaneously labeled with a cellular barcode sequence and also receives a universal amplification handle. The incorporation of these two features is a result of template-switching events. Consequently, template switching is the core of the approach. Following the barcoding, all reactions can be pooled and from this step processed in a single reaction tube. The reverse transcription / template switching and pooling are followed by PCR amplification where the cDNA molecules are amplified using a single universal primer. The obtained full-length cDNA library is then reformatted to an Illumina sequencing library using standard methods. In addition to the streamlined procedure, STRT retains strand information and, moreover, the template-switching event occurs predominantly at the 5´-ends of transcripts meaning that the position of the transcription start site (TSS) can be obtained. 

Several studies have explored the parameters of and the conditions for template switching. Some investigations have focused on the nature and number of the incorporated nucleotides. For instance, it was demonstrated that supplementing the reaction with manganese ions increased the number of incorporated nucleotides from a single residue to 3-4 [12]. Different MMLV reverse transcriptases, predominantly SuperScript II (SSII; Life / Invitrogen) and SuperScript III (SSIII; Life / Invitrogen), have been examined for their template-switching proficiency [7] [13,14]. The TSO has also been studied. It has, for example, been shown that 5´-modifications of this secondary oligonucleotide can minimize the formation of products carrying tandem copies of the TSO [15]. 3´-blocking of the TSO has been demonstrated to eliminate spurious priming of the TSO during cDNA synthesis and PCR amplification [16]. Finally, the artifacts generated by the template-switching mechanism have also been the subject of research. A recent study put forward the process of strand invasion whereby premature template switching, at the positions where the sequences of the RNA molecule and the 3´-part of the TSO are the same, generates truncated, but correctly tagged, cDNA molecules [17]. 

In this article, we use fully degenerate TSOs to directly determine the base preference of the terminal transferase activity of SuperScript II. In addition, we report careful optimizations of all the relevant parameters in the reaction, which leads to an integrated set of guidelines for efficient template switching in the context of cDNA amplification from single cells. The STRT method served as the framework for the analyses. However, as has been described above, template switching is incorporated into a number of RNA seq protocols, and therefore the results of this work are applicable beyond STRT.

## Materials and Methods

The scope of the investigations presented in this article necessitated a variety of experimental setups. However, all experiments were based on STRT and shared common features. Therefore, this section begins with a general description of the templates used, of the primers and oligonucleotides, of the STRT protocol and of the readout with quantitative real-time PCR (qPCR). These descriptions are followed by the precise details (reagents, conditions and parameters) for each individual experiment. As a number of experiments featured readout with massively parallel Illumina sequencing, an overview of the sequencing library preparation from double-stranded cDNA is also provided.

### Template

Two types of templates were employed in this study: total RNA and synthetic RNA spikes. 1 μl of either a 1 ng/μl or 10 ng/μl of total RNA was used. This RNA was either universal human reference RNA (Agilent / Stratagene; Cedar Creek, TX, USA) or total RNA from the mouse ES cell line R1 (ESR1). ERCC RNA spike-in controls (Life / Ambion; Carlsbad, CA, USA) served as the synthetic RNA molecules. 1 μl of a 1:1000 dilution of the original ERCC spike concentration was used. This translates into a total of about 62.3 million molecules or 104 attomoles. Accordingly, the spike concentration in a 10 μl reaction was 10.4 pM.

### Primers and oligonucleotides

The sequences of all employed primers and oligonucleotides are presented in Table S1 in File S1.

Oligo dT was used to prime first-strand synthesis. It was 5´-biotinylated and was a combination of the STRT amplification handle, a recognition sequence of SalI and a stretch of 30 thymidine residues. Additionally, it had a 3´-VN anchor to align it with the start of the transcript. The oligo dT – denoted STRT Bio-T30VN – was ordered from Eurofins MWG Operon (Ebersberg, Germany).

In all experiments the template-switching oligonucleotide (TSO) carried the STRT amplification handle, a barcode (either degenerate or fixed) of varying length and three ribo bases (either degenerate or guanosines). In addition, some of the TSOs incorporated the recognition site for BtsI. Moreover, in some cases uracil was substituted for thymidine residues to enable a subsequent uracil excision and thus a TSO removal. The sequences of the different TSOs are shown in Table S1 in File S1. The TSOs with fixed content were ordered from Eurofins MWG Operon, as were the TSOs having degenerate positions for the TSO length experiment. The remaining TSOs with degenerate positions (used in the sequenced experiments) were synthesized by Integrated DNA Technologies with the “hand-mix” option for the random positions (Coralville, IA, USA). With this option enabled a bottle of nucleotides is hand mixed and placed on a fifth port on the synthesizer. According to IDT this “will ensure a 1:1:1:1 ratio” for the oligonucleotides.

The PCR amplification primer – STRT-PCR – had the STRT universal amplification sequence and was 5´-biotinylated. It was purchased from Eurofins MWG Operon.

### General STRT protocol

Firstly, a cell capture mix (CCM) was prepared. The standard mix contained 0.4 μM STRT Bio-T30VN (oligo dT) and 2 μM template-switching oligonucleotide (TSO) in 1x STRT buffer (20 mM Tris-HCl [pH 8.0], 75 mM KCl, 6 mM MgCl_2_ and 0.02% Tween-20 [Merck; Darmstadt, Germany]). The different experiments featured slightly different cell capture mixes. Especially the TSO sequences and amounts were varied. The details of each experiment are described in the subsequent sections. 5 μl of the CCM were deposited in a well of an 8-well PCR strip kept on ice. Subsequently, 1 μl of template was added and the strip stored at -20°C.

Prior to reverse transcription and template switching the strip was thawed by placing it in a thermocycler (MJ Research PTC-200 now a part of Bio-Rad; Hercules, CA, USA) at 20°C for 5 min. Thereafter, 4 μl of a reverse transcription mix were added. This mix contained 5 mM DTT, 2.5 mM dNTPs, 7.5 mM MnCl_2_ and 20 units of SuperScript II (Life / Invitrogen; Carlsbad, CA, USA) in a 1x STRT buffer without magnesium chloride. Accordingly, the final 10 μl reaction contained 1 μM of TSO, 0.2 μM of oligo dT, 1 mM dNTPs, 2 mM DTT, 3 mM MgCl_2_, 3 mM MnCl_2_ and 20 units of SSII. The reaction was placed in a thermocycler with a temperature profile consisting of 10°C for 10 min and 42°C for 45 min. Afterwards, it was immediately placed on ice.

A bead-based procedure was then employed to purify the template-switched first-strand cDNA. For each reaction, 2 μl of Dynabeads MyOne carboxylic acid (Life / Invitrogen) were washed twice with an equal volume of EBT (EB [Qiagen; Hilden, Germany] supplemented with 0.02% Tween-20 [Merck]) and resuspended in 1 μl of EBT. Subsequently, 20 μl of 14% PEG-6000 (BDH Laboratory Supplies; Poole, England) in 0.9 M NaCl were added to the resuspended beads. When several reactions were purified simultaneously the volumes given above were scaled up accordingly. The PEG concentration was set at 14% as this percentage gives a purification cutoff between 100 and 150 bases [18]. As such, cDNA molecules longer than 100 bases were efficiently captured and recovered. The bead-solution in PEG was added to the reverse transcription / template-switching reaction and the cDNA products immobilized at RT for 20 min. The beads were collected with a magnet (in a magnetic particle concentrator), the pellet washed twice with freshly-prepared 70% ethanol and allowed to dry. The drying was performed by removing the lids and allowing the reaction vessels to stand at RT for about 10 min. Finally, the immobilized cDNA was eluted in 37 μl of EB. In case amplification was not performed immediately, the eluted products were placed in -20°C.

### qPCR

The reaction outcome was analyzed with quantitative real-time PCR (qPCR). 3 or 5 μl of the eluted template was combined with 0.2 mM dNTPs, 200 nM of the STRT-PCR primer, 0.5x SYBR Green I (Life / Invitrogen) and 1x Advantage 2 polymerase mix (Clontech; Mountain View, CA, USA) in 1x Advantage 2 PCR buffer (40 mM Tricine-KOH [pH 8.7], 15 mM KOAc, 3.5 mM Mg(OAc)_2_, 3.75 μg/ml BSA, 0.005% Tween-20 and 0.005% Nonidet-P40; Clontech). The total reaction volume was 10 μl. The reaction was cycled in a 7900HT Fast real-time PCR instrument (Life / Applied Biosystems) with the following parameters: enzyme activation at 95°C for 1 min, 35 cycles of denaturation at 95°C for 30 s, annealing at 65°C for 30 s and extension at 68°C for 4 min. After completion of the cycling stage, a dissociation stage was implemented. Here, the temperature was raised in 0.5°C increments from 60°C to 95°C while monitoring the SYBR Green signal.

The obtained amplification and dissociation curves were analyzed with Sequence Detection System (SDS) version 2.3 (Life / Applied Biosystems) and the obtained threshold cycle (Ct) values exported and further analyzed in Microsoft Excel (Microsoft, Redmond, WA, USA).

### TSO concentration

For the TSO concentration experiment, the STRT v2-7 TSO was used. A CCM concentration of 2 μM was compared with 20 nM, 80 nM, 400 nM, 5 μM and 10 μM (the corresponding concentrations during template switching were half of the concentrations in the CCMs). 400 nM of STRT Bio-T30VN was used as oligo dT. 1 ng of universal human total RNA constituted the template. Triplicate reactions were performed. Singlicate reactions were carried out for the no template control (NTC), containing water instead of total RNA.

### TSO length

For the length experiment, a set of progressively longer TSOs, based on STRT v2-7 but omitting the BtsI recognition sequence, was designed and ordered from Eurofins MWG Operon (Table S1 in File S1). These TSOs had degenerate nucleotide stretches ranging from two to 12 positions. Accordingly, the lengths for the different TSOs were 35 (STRT v2-7-N2), 37 (STRT v2-7-N4), 39 (STRT v2-7-N6), 41 (STRT v2-7-N8), 43 (STRT v2-7-N10) and 45 (STRT v2-7-N12). Separate reactions with a CCM concentration of 2 μM were set up. 400 nM of STRT Bio-T30VN was used as oligo dT. The template used was 1 ng of ESR1 total RNA. Triplicate reactions were carried out. 

### Reverse transcriptase

SuperScript II (SSII; Life / Invitrogen), SuperScript III (SSIII; Life / Invitrogen) were assessed in triplicate reactions entailing 1 ng of ESR1 total RNA. For SSII, 20 units of enzyme were used with a temperature profile of 10°C for 10 min and 42°C for 45 min. 20 units of SSIII were employed with a temperature profile consisting of 10°C for 10 min, followed by 50°C for 45 min. Furthermore a cycled SSIII reaction was performed – 8 cycles of 50°C for 5 min and 60°C for 1 min – based on the work of Carninci et al. [19]. 2 μM of STRT v2-7 served as the TSO. 400 nM of STRT Bio-T30VN was employed to prime the cDNA synthesis. The template was 1 ng of ESR1 total RNA. In the no template control (NTC) reactions, performed in singlicate, total RNA was replaced by water.

### Amount of SuperScript II

For determination of the optimal SSII amount, four reactions were set up according to the conditions outlined above, except that 1 ng of universal human total RNA was employed. The four reactions encompassed 1 unit, 5 units, 10 units, 50 units and 200 units of the enzyme. Triplicate reactions were carried out. Singlicate reactions were implemented for the no template control (NTC), containing water instead of total RNA.

### Sequence analysis at the template-switching junction

To fully investigate the sequence preferences at the template-switching junction as well as the composition and number of bases incorporated by the reverse transcriptase in a template-independent fashion, a set of reactions encompassing TSOs with degenerate positions was processed and sequenced. The combination of template and TSO for these reactions is outlined in Table 1.

**Table 1:** | Summary of sequenced libraries.

**Reaction**	**Template**	**TSO**	**Number of reads**
ERCC10G3	ERCC Spike-in controls	STRT N10-rG3	260 374 806
ERCC10G3	ERCC Spike-in controls	STRT N10-rN3	245 507 832
RNA10G3	Universal human reference RNA	STRT N10-rG3	243 984 371
RNA12G3	Universal human reference RNA	STRT N12-rG3	230 561 931
RNA10N3	Universal human reference RNA	STRT N10-rN3	104 772 398

The reactions performed to determine sequence preferences at the template-switching interface and the sequencing statistics.

Three TSOs with degenerate positions immediately upstream of the ribo base portion were designed:

• STRT handle – 10 degenerate DNA positions – 3 degenerate RNA positions
• STRT handle – 10 degenerate DNA positions – 3 riboG positions
• Truncated STRT handle – 12 degenerate DNA positions – 3 riboG positions

The reason for the two-base truncation of the TSO with the longest degenerate stretch was to keep the total lengths of the TSOs equal. These oligonucleotides were ordered from Integrated DNA Technologies and their precise sequences are given in Table S1 in File S1.

Two sets of reactions were performed. One set used approximately 62.3 million in vitro transcribed ERCC spikes (104 amol; Life / Ambion). The other set encompassed 10 ng of universal human reference RNA (Agilent / Stratagene).

The exact TSOs and templates used in the performed reactions are outlined in Table 1.

The reverse transcription / template switching and clean-up were performed as described in the ‘General STRT protocol’ section. Firstly, a qPCR was performed to determine the optimal number of cycles for the PCR amplification reaction to be processed. 3 μl of the purified sample entered the qPCR protocol as described in previous sections. 32 μl were subsequently used in the actual full-length cDNA amplification. The amplification parameters and conditions were the same as outlined above, except that the final volume was 50 μl (instead of 10 μl), a 15 s denaturation was performed (instead of 30 s), SYBR Green was omitted and that USER enzyme was employed to digest the TSO to prevent it from priming during the amplification. In particular, the 49 μl uracil removal reaction contained 5 units of USER enzyme (New England Biolabs; Ipswich, MA, USA) and was incubated at 37°C for 15 min in a thermocycler (MJ Research PTC-200) and then placed on ice. 1 μl of 50x Advantage 2 polymerase mix (Clontech) was then added to finalize the 50 μl amplification that encompassed 0.2 mM dNTPs, 200 nM of the STRT-PCR primer, 5 units of USER enzyme (New England Biolabs) and 1x Advantage 2 polymerase mix (Clontech) in 1x Advantage 2 PCR buffer (40 mM Tricine-KOH [pH 8.7], 15 mM KOAc, 3.5 mM Mg(OAc)_2_, 3.75 μg/ml BSA, 0.005% Tween-20 and 0.005% Nonidet-P40; Clontech). This was cycled in a PTC-200 thermocycler (MJ Research) according to the following profile: activation at 95°C for 1 min, n cycles of denaturation at 95°C for 15 s, annealing at 65°C for 30 s and extension at 68°C for 4 min. Here, n is the optimal number of cycles based on the qPCR run and ranged from 12 to 19 in this experiment. Following the cycling, 5 μl of the amplification were transferred to a new tube containing 45 μl of amplification mix (without USER) and amplified for five additional cycles. The outcome of the latter reaction was run on a 1.2% agarose gel with SYBR Safe (Life / Invitrogen) in an E-Gel electrophoresis system (Life / Invitrogen). This was performed as a control of the amplification.

25 μl of the amplification product (i.e. half of the reaction) was immobilized on 20 μl streptavidin-coated beads (Dynabeads MyOne Streptavidin C1; Life / Invitrogen) and processed to a final sequencing library according to the previously published protocol [11] with the difference that the SalI digestion and ADP2 ligation were performed separately with immobilization and three washes with 50 μl EBT in-between. Briefly, the immobilized sample was fragmented using 5 μl of NEBNext dsDNA Fragmentase (New England Biolabs) at 37°C for 45 min. The bound fragments (the 5´- and 3´-fragments of the amplified ds cDNAs carrying a terminal biotin) were thoroughly washed and their ends repaired using the NEBNext End Repair system (New England Biolabs). The polished fragments were then A-tailed using the NEB Next dA-tailing module (New England Biolabs). A SalI digestion using SalI HF (New England Biolabs) was carried out to cut, and thus to release, the 3´-fragments. An adaptor was ligated to the immobilized A-tailed 5´-fragments using T4 DNA ligase (Thermo Fisher Scientific / Fermentas; Burlington, Canada). The dual-adaptor carrying products (STRT handle on one end and the ligated adaptor on the other) were then PCR amplified using 2 units of Phusion polymerase (Thermo Fisher Scientific / Finnzymes; Vantaa, Finland) in a 100 μl reaction (divided into two 50 μl reactions) containing 1 μM of forward primer (carrying the STRT handle fused to one of the Illumina adaptors; for sequence see 11), 1 μM of reverse primer (encompassing the ligated adaptor sequence fused to the other Illumina adaptor; for sequence see 11), 200 μM dNTPs in 1x Phusion HF buffer (Thermo Fisher Scientific / Finnzymes). The cycling was performed in a PTC-200 thermocycler (MJ Research) with enzyme activation at 98°C for 30 s, 14 cycles of denaturation at 98°C for 10 s, annealing at 65°C for 30 s and extension at 72°C for 30 s and a final elongation at 72°C for 5 min. The amplified products were purified with Agencourt AMPure XP beads (Beckman Coulter; Indianapolis, IN, USA) using the recommended 1.8x ratio of beads to PCR reaction. The products were finally eluted in 40 μl EBT buffer. Products of between 200 and 600 bp were selected by excising the corresponding region from a 2% agarose gel with SYBR Safe (Life / Invitrogen) run in an E-Gel system (Life / Invitrogen) and purifying the fragments from the gel slice using the QIAquick Gel Extraction kit (Qiagen). Manufacturer’s recommendations were followed, except that the gel was dissolved by 15 min agitation instead of by heating. The quantity of the final library was assessed using the Qubit dsDNA HS kit (Life / Invitrogen) in the Qubit 2.0 fluorometer (Life / Invitrogen).

Each library was sequenced on a single lane of a HiSeq2000 instrument (Illumina; San Diego, CA, USA) using TruSeq v3 chemistry (Illumina). The molarity loaded on the instrument was 12 pM. The reads passing the built-in quality filters were subsequently analyzed.

### Sequencing data analysis

The reads passing the built-in Illumina quality filters were queried for the transcript and spike sequences of interest with the *grep* command in the Unix environment. The sequencing reads matching the query sequences were piped into text files. Generally, sequences from the 5´-ends of transcripts and spikes were used. The human transcript sequences were derived from definitions in the *refFlat.txt* file for hg19 from the UCSC Genome Browser. The spike sequences were taken from information provided by the manufacturer (Life / Ambion). All queried sequences are provided in Table S3 in File S1. The first 500 entries from each resultant text file were visualized in WebLogo 3 (http://weblogo.threeplusone.com/create.cgi) for verification purposes. Additionally, the hit distributions along the analyzed human transcripts were analyzed using the UCSC Genome Browser (Figure S2 in File S1).

For the reactions entailing human total RNA, five transcripts were evaluated: MALAT1 (metastasis associated lung adenocarcinoma transcript 1; a non-coding transcript), RPLP1 (ribosomal protein, large, P1), MT2A (metallothionein 2A), AHSG (alpha-2-HS-glycoprotein) and CNIH4 (cornichon homolog 4). These transcripts spanned a range of expression levels. For the experiments featuring spiked-in RNA, the two most highly “expressed” spikes – MC28 and MJ-500-37 – were analyzed. 

The counts for the transcripts and spikes of interest were obtained by calculating the number of reads in each of the output files. The bases and sequences at the template-switching junction, as well the various barcode sequences upstream of the template-switching site, were extracted and counted using custom scripts implemented in Perl and BioPerl. These scripts are available upon request. All reads participated in the analyses, except for the complexity comparison between RNA10G3, RNA12G3 and RNA10N3, as well as ERCC10G3 and ERCC10N3, where the number of reads was normalized to preserve the expression level. UMIs were processed as previously described [11]. Further analysis and visualization was performed in Microsoft Excel.

Raw sequence data is available in the NCBI Sequence Read Archive (accession SRP028287).

## Results and Discussion

We have evaluated the optimal conditions for reverse transcription, and the base preferences of the terminal transferase activity of RT, with a view to finding the most efficient conditions for cDNA amplification from single cells. STRT, our high-throughput RNA-seq method for single cells, has served as the framework for these optimizations [10]. Two principal readout platforms were used throughout. First, cDNA yield was monitored under varying conditions by amplifying the full-length cDNA in a quantitative real-time PCR (qPCR) instrument. Second, the sequence motifs generated at the template-switching interface were monitored by sequencing a number of full-length cDNA libraries on an Illumina HiSeq2000 instrument (Table **1**). This offered a base-resolution view of the region where template switching occurs, allowing us to draw conclusions about sequence motif preferences.

### TSO concentration

**Figure 1:**
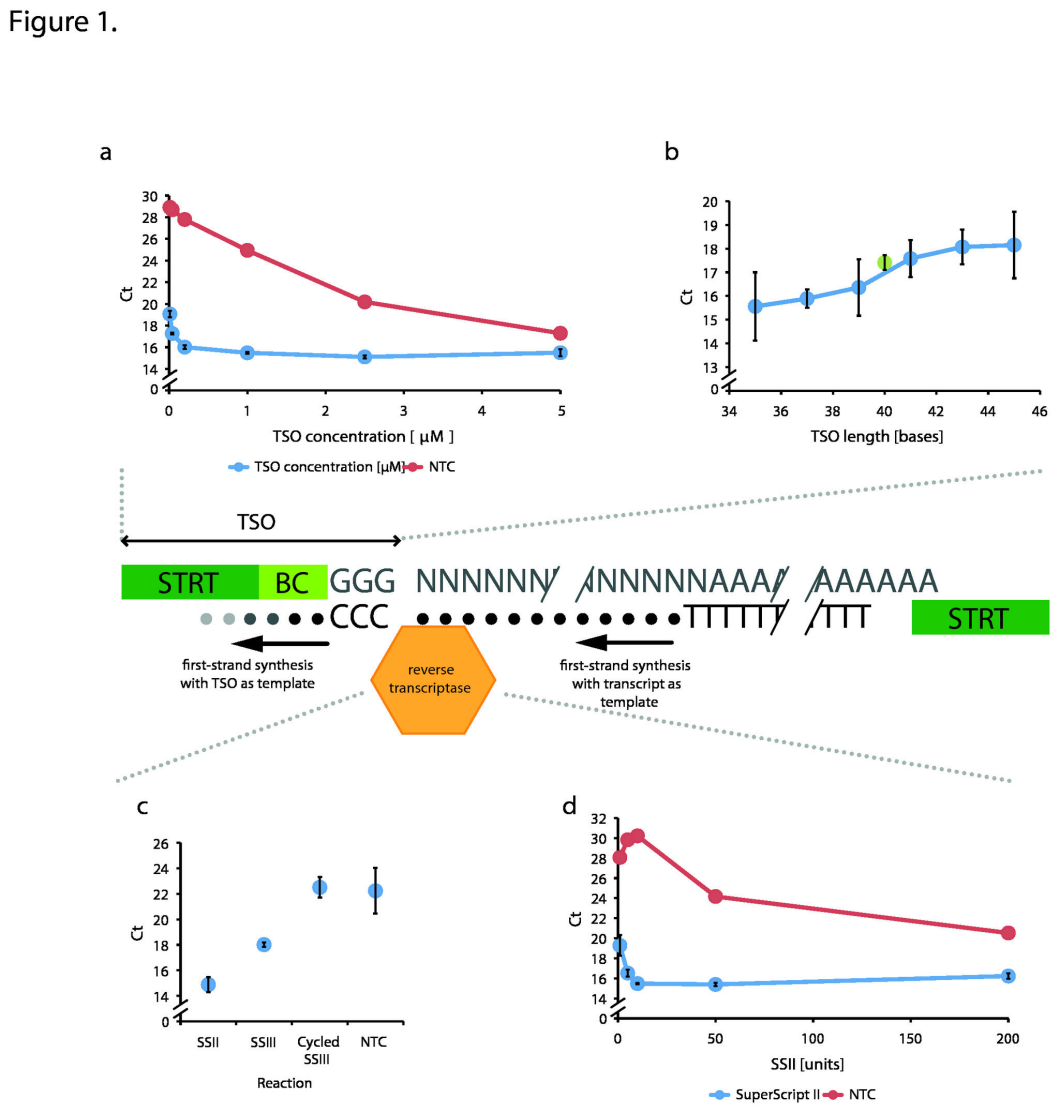
| Optimal conditions for template switching. The qPCR-derived Ct values for the investigated conditions are shown. Please note that the y-axis scales are broken and that the graphs have different Ct value scales. Also, no error bars are shown for the no template controls (NTC) as these reactions were performed in only one tube. In contrast, the actual experiments were performed in triplicate with the standard deviation error bars displayed in the graphs. The central part of the figure illustrates the template-switching process in the setting of our STRT method. (a) The optimal TSO concentration was 1 μM and (b) shorter TSOs showed a tendency for better performance (see also Table **S2** and Figure **S1** in File S1). The green datapoint in the TSO length graph represents the published version of the TSO [10] that is 40 bases in length. (c) shows analysis of different RT enzymes. SSII was a better choice than SSIII and, a cycled SSIII protocol (8 cycles of 50°C for 5 min and 60°C for 1 min; [19]) did not work, possibly due to the elevated temperature periods inactivating the enzyme (see also Table **S2** in File S1). (b) The optimal SSII amount was 10 units per 10 μl reaction.

Both the original SMART kit (Clontech) and more recent protocols, such as SMARTer Ultra Low RNA kit (Clontech PT5163) use a TSO concentration of 1.2 µM, while the NanoCAGE protocol uses 10 µM [13]. In contrast, the original STRT protocol used 200 nM. We compared cDNA yield using the original 200 nM concentration as well as 10 nM, 40 nM, 1 µM, 2.5 µM and 5 µM (Figure **1a**). The two lowest concentrations gave inferior results compared to the original 200 nM concentration (p<0.001 in both cases, Table S2 in File S1). In contrast, the intermediate concentrations of 1 and 2.5 µM produced better results at a high statistical significance (p<0.01, Table **S2** in File S1). Finally, no significant difference was found between 5 µM and 400 nM (Table **S2** in File S1). However, since the no template control (NTC) reactions produced lower Ct values at higher TSO concentrations (assuming the formation of spurious and unwanted products [20]), we conclude that the optimal TSO concentration was 1 µM. 

### TSO length

The SMART kit (Clontech) employs a 39-base TSO. In the subsequent SMARTer kits this length has been shortened to 30 bases (SMARTer Ultra Low RNA kit; Clontech PT5163), hinting at that shorter TSOs might be beneficial. The NanoCAGE approach uses an even shorter TSO, 27 residues in length [13]. In contrast, the TSO in the original STRT was 40 bases long [10]. 

In order to evaluate the effect of TSO length on template switching efficiency, we designed a set of TSOs based on the STRT TSO but varying in length between 35 and 45 bases. As expected, we found that shorter TSOs resulted into higher template-switching efficiencies (Figure **1b**). The coefficient of determination (R^2^) was 0.94, signifying a good linear fit of the data (Figure **S1** in File S1). Thus, in the range examined, shorter TSOs are preferable. 

### Reverse transcriptase

A number of template switching protocols have used SuperScript II (SSII; Life / Invitrogen; [7,21]), an MMLV derivative. However, this enzyme has its maximum activity at only 42°C and may not be able to pass stretches of RNA with secondary structure. An RT with increased thermostability might be a better alternative as the secondary structures are melted to a higher extent allowing efficient extension. One such thermostable enzyme is SuperScript III (SSIII), an engineered mutant of SSII with an optimal reaction temperature of 50°C (Life / Invitrogen). Although some reports suggest it does not perform template switching [7], others have found that it does [13] [14]. 

We compared SSII with SSIII using qPCR readout. In addition, the constant elevated temperature for SSIII (50°C for 45 min) was compared to a cycling protocol based on the work of Hayashizaki and colleagues [19]. In the cycling protocol, the temperature was raised from 50°C to 60°C for 1 min, to further resolve potentially impairing secondary structures. Eight such cycles (50°C for 5 min and 60°C for 1 min) were implemented. We found that SSII performed consistently better than SSIII (Figure **1c**). The difference was statistically significant (p=0.018, Table **S2** in File S1). The constant-temperature SSIII procedure was successful in carrying out template switching as evidenced by an amplification signal separate from the negative control. The efficiency was, nevertheless, lower compared to SSII. On the other hand, the cycled SSIII protocol was indistinguishable from the negative control. One plausible explanation for the failure of this protocol is inactivation of the RT by the raised temperature. 

### Amount of SuperScript II

Next, different amounts of SSII were evaluated. The original STRT protocol used 20 units of SSII per 10 μl reaction[10]. Most of the competing techniques use 100 to 200 units of RT ([13], Clontech SMART and SMARTer kits). Here, total SSII amounts of between 1 and 200 units were assessed. We observed a Ct value curve with an elongated U shape where intermediate SSII amounts produced the best results (Figure **1d**). We found that 10 and 50 units were significantly better than the other amounts (p<0.01 for all comparisons except for 50 U vs. 200 U where p=0.011, Table **S2** in File S1). As higher SSII amounts tend to produce more background, evidenced by the no template control (NTC) values, we conclude that 10 units of SSII was the best choice. 

### Sequence preferences at the template-switching junction

Having found optimal conditions for the reaction, we next used fully degenerate TSOs to probe the non-templated nucleotides added by reverse transcriptase. By sequencing the template-switching region with the massively parallel Illumina HiSeq2000 instrument and analyzing the millions of obtained reads, a detailed view of the template-switching junction could be obtained, shedding light on fundamental aspects of this process.

In order to comprehensively study the sequence preferences at the template-switching site five reactions were performed and sequenced (Table 1). The employed TSOs carried degenerate stretches at the template-switching position. Either ERCC spike-in RNA (62.3 million molecules; Life / Ambion) or universal human reference RNA (10 ng; Agilent / Stratagene) served as template. A detailed description of the experiments is provided in the materials and methods section. The sequencing yields are shown in Table **1**. The RNA10N3 reaction, producing weak gel smears during the library preparation, gave about 105 million reads. On average, 245 million reads were obtained for the four remaining reactions.

For the experiments featuring spiked-in RNA, the two most abundant spikes – MC28 and MJ-500-37 – were analyzed (Table 2). Spike MC28 exhibits 67 million reads in ERCC10G3 and 46 million in the ERCC10N3 reaction, respectively. The analogous figures for spike MJ-500-37 were 16.1 million and 16.2 million, respectively.

**Table 2:** | Read numbers for RNA spikes analyzed.

**Reaction**	**MC28**	**MJ-500-37**
ERCC10G3	67 025 156	16 124 820
ERCC10N3	45 974 543	16 214 015

For the reactions with human total RNA, five different transcripts were evaluated: MALAT1 (a non-coding transcript), RPLP1, MT2A, AHSG and CNIH4. These five transcripts span a wide range of expression levels (Table **3**). For example, in the three reactions with total RNA, MALAT1 generated 4.2 million reads in total, whereas CNIH3 produced only about 7000.

**Table 3:** Read numbers for transcripts analyzed.

**Reaction**	**MALAT1**	**RPLP1**	**MT2A**	**AHSG**	**CNIH4**
RNA10G3	2430437	518171	160475	44197	3391
RNA12G3	1560168	366436	116468	26898	2170
RNA10N3	206272	224778	75713	20059	1411
**Total**	**4196877**	**1109385**	**352656**	**91154**	**6972**

Template switching can occur at multiple locations along the transcript. It should be emphasized that here, only the major template-switching site was taken into consideration. This was done to eliminate sequence differences on the transcript side of the template-switching site when investigating the bases introduced by the RT and the sequence preferences on the TSO side. The major template-switching peak accounted for between 60.0% (AHSG) and 94.5% (RPLP1) of all reads of that particular transcript in the RNA10G3 reaction. The major sites for all transcripts, except MALAT1, occurred at the 5´-end indicating that RT has extended the full length of the transcript before adding the extra bases necessary for annealing of the secondary oligonucleotide (**Figure S2 **in File **S1**). For MALAT1, the main template-switching peak occurred about 1300 bases from the 5´-end. However, this noncoding transcript has some interesting and unusual properties, which may explain this departure from the 5´-end. Besides being broadly expressed throughout human tissues, MALAT1 possesses a 3´-end processing mechanism by which both a nuclear-localized long noncoding species and a cytoplasmic small RNA are generated [22]. The presence of a peak at 1.3 kb was also consistent with CAGE data available on the UCSC and FANTOM genome browsers.

The expression levels of the investigated transcripts, based on the number of reads, generally showed a high degree of correlation. For example, the R^2^-value between the RNA10G3 and RNA12G3 experiments was >0.99 (**Table S4 **in File **S1**). MALAT1 generated a low number of reads in the RNA10N3 reaction and was therefore removed in this analysis. The fact that MALAT1 was underexpressed in the RNA10N3 reaction may once again be attributed to this noncoding transcript having special characteristics, as described above. In addition to these consistent expression levels, the distributions of the sequencing reads along the transcripts were highly similar (Figure **S2** in File S1).

### Base composition of the template-independent incorporation

The nontemplate nucleotide addition, performed by the RT upon reaching the end of the template molecule, is an important event necessary for template switching. To study the base composition of the template-independent incorporation, we analyzed the bases immediately upstream of the template-switching site in the sequencing reads. Given the assumption that the bases at the corresponding positions of the TSO participate in Watson-Crick pairing with the bases added to the cDNA in a nontemplate manner by the RT, reading the sequencing reads at these positions reveals the nature of the RT-added nucleotides. For this analysis, the reactions featuring TSOs with fully degenerate ribo base stretches were employed as these include all possible sequence combinations and thus allow for perfect matches with the non-templated nucleotides.

**Figure 2:**
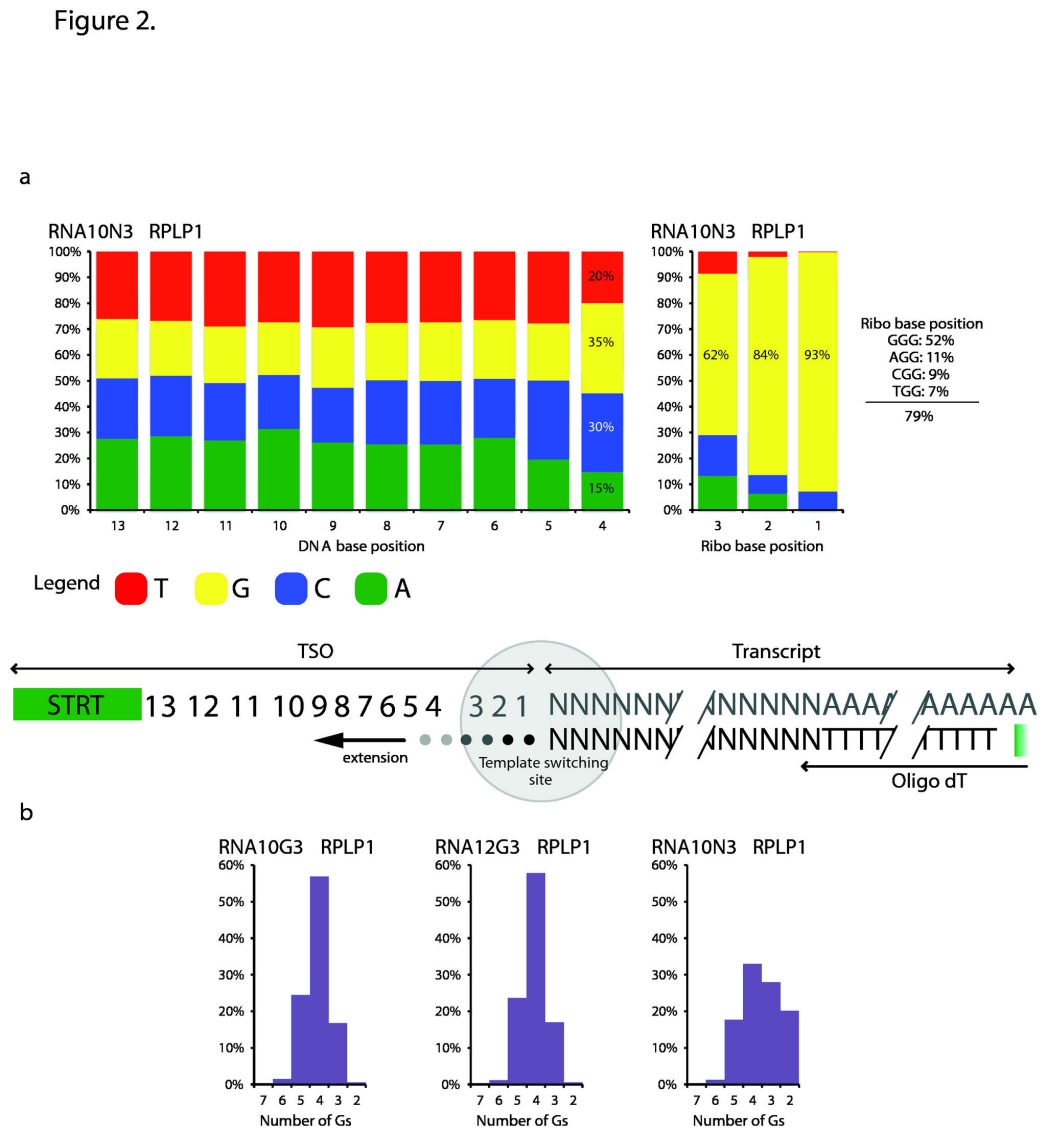
| Optimal TSO sequences for template switching. The center panel shows the template-switching process. The template-switching event occurs in the middle of the grey circle. The investigated positions of the TSO are numbered from the template-switching site. The first three positions correspond to ribo bases and are shown in grey. The other analyzed positions are DNA bases and are depicted in black. In (a)) nucleotide preferences for all positions for RPLP1 in the RNA10N3 sample are shown. Corresponding graphs for the other transcripts and RNA spikes are shown in Figures **S3** and S4 in File S1. (b)shows the distribution of the number of guanidines seen in the sequencing output for RPLP1 in the three performed reactions. Corresponding graphs for the other transcripts and RNA spike molecules are shown in Figure **S5** in File S1. The numbers of reads for the analyzed RNA spikes and transcripts are shown in Table **S6** and Table **S7** in File S1, respectively.

For both the reaction employing ERCC spikes and total RNA, a clear trend with a strong preference for guanosines in the ribo base portion of the TSO was observed (Figure **2a**, Figure **S3** and Table **S5** in File S1). This preference was most pronounced at the 3´-position, i.e. in the position directly at the template-switching junction. The guanosine frequency was reduced with increasing distance from the template-switching site.

For ERCC spike MC28 the guanosine percentages for the three ribo base positions, starting with the 3´-most position, were 98%, 80% and 46% (Figure **S3** and Table **S5** in File S1). Similar values were obtained for spike MJ-500-37 (Figure **S3** and Table **S5** in File S1). Similarly, for the endogenous transcript RPLP1, the guanosine percentages in the three ribo base positions were 93%, 84% and 62%. MALAT1 and MT2A produced comparable results (Figure **2a**, Figure **S3** and Table **S5** in File S1). 

Taken together, the average percentages for guanosine in the three ribo base positions were 94%, 83% and 57% (Figure **S3** and Table **S5** in File S1). GGG corresponded to 46% of the reads, followed by AGG with 14%, CGG with 11% and TGG with 8%. 

In summary, there was a strong preference for cytosine addition by the reverse transcriptase. However, other bases can also be incorporated, albeit at a lower frequency. With increased distance from the end of the transcript, the preference for cytosine decreases. This has consequences for the design of optimal template switching oligos. All current protocols use TSOs with a GGG motif, which would be complementary to only 46% of all cDNA molecules. Simply replacing this with an NGG motif would make it possible to capture 79% of all cDNA molecules. However, it should be kept in mind that TSOs with random positions imply a lower effective concentration of each individual TSO. Additionally, the increased TSO complexity caused by the degenerate position in the ribo base region might increase the number of artifacts due to mispriming events. 

### Preferred sequence of DNA bases upstream of the ribo section in the TSO

Next, we expanded the above analysis to the ten DNA bases located directly upstream of the ribo portion of the TSO. There was a clear preference for guanosine in the DNA position immediately preceding the ribo base stretch (Position 4; Figure **2a**, Figure **S4** and Table **S6** in File S1). For all analyzed cases, except MALAT1 in the RNA10N3 reaction, guanosine was the preferred nucleotide with percentages above 30%. For MALAT1 in the RNA10N3 reaction, G accounted for 32% and C for 33% at this position. The guanosine frequencies ranged from 31% (spike MC28 in ERCC10N3) to 40% (RPLP1 in RNA12G3).

As cytidine has been demonstrated to be the preferred residue for RT to incorporate during the template-independent addition, and, as will be shown in the next section, the introduction of several cytidines is a frequent event, an extra guanosine immediately preceding the ribo bases in the TSO provides this oligonucleotide with an additional annealing point, enhancing template switching. This also explains the differences in efficiency observed among different barcodes in the original STRT protocol: barcodes ending in guanosine were more efficient because this guanosine was placed at position 4, and thus participated in the template switching mechanism (data not shown).

It is more difficult to draw conclusions about the bases further away from the template-switching site. In general, for the two next positions, the distribution of the nucleotides approached background with each of the bases having a frequency of about 25% (Figure **2a**). Further upstream, there was a very slight tendency for adenosine and thymidine preference (Figure **2A**). The fact that all these positions showed frequencies so close to 25% also confirms that the oligonucleotides synthesis was nearly unbiased, which in turn supports our interpretation that the guanine preferences closer to the template-switching site were not due to a synthesis bias.

### Number of residues introduced in a template-independent manner

We next examined the number non-templated nucleotides added by the reverse transcriptase. Counting the number of guanosines at the template-switching interface in the sequencing data provides an estimate of the number of bases introduced. However, from the sequencing output it is impossible to differentiate between guanosines originating from cytidine addition by the RT and guanosines present at the 3´-end of the TSO. For example, if three guanosines are found at the template-switching junction in a sequencing read, two of these might be derived from cytidine addition by the RT (these residues thus participated in the annealing between the TSO and cDNA) with the last base being a remnant of a guanosine present at the 3´-end of the TSO. However, it is equally possible that the enzyme added all three residues or that the RT added a single nucleotide and the two remaining guanosines originated from the TSO. As we in this analysis also counted guanosines in the 3´-DNA base (i.e. position 4) and in the ribo bases of the TSO (i.e. positions 1 to 3), our data provides an upper estimate of the number of bases added.

Both spike MC28 and MJ-500-37 start with the sequence GGAATTCT. Therefore, we counted the number of instances of the motif G(n)GGAATTCT, where n was between two and eight. Over 95% of the corresponding reads contained the motif. For MC28 in ERCC10N3 two to five guanosines were most frequently observed with G(3) being the most prominent (36%: Figure **S5** and Table **S6** in File S1). Similar figures were obtained for MJ-500-37 in ERCC10N3. Also in this case, three guanosines were most frequently observed (32%). For the ERCC10G3 reaction, four and five guanosines were predominantly found (Figure S5 and Table S7 in File S1). For MC28 the percentages were 63% for G(4) and 28% for G(5). In the case of MJ-500-37 60% had four guanosines and 29% had five. In this case the TSO already included three guanosines, so that fewer than three guanosines could only be observed due to sequencing errors, or errors in TSO synthesis.

To expand on this analysis, a similar investigation was performed for RPLP1 and MT2A in the three reactions with total RNA (Figure **2b**, Figure **S5** and Table **S7** in File S1). As the RPLP1 template-switching site starts at CCTTTCCT, we searched for the motif G(n)C(2-4)TTTCCT, with n being between two and eight. The number of cytidines was kept flexible between two and four to allow for sequencing error and thus for inclusion of more reads. Similarly, as the TS position for MT2A starts with the sequence ACCACGCC, the motif G(n)ACCACGCC was queried. Also here, n was between two and eight. The obtained results were analogous to the situation with the ERCC spikes. For the RNA10N3 reaction, two to five guanosines were predominating for both RPLP1 and MT2A, with three guanosines observed most frequently. Three to five guanosines were most frequently observed for the RNA10G3 and RNA12G3 reactions. 

Not surprisingly, for both ERCC spikes and transcripts, the frequency declined with the length of the guanosine stretch (Figure **S5** and Table **S7** in File S1). For six guanosines the percentages ranged from 0% (MT2A in RNA10N3) to 4% (spike MC28 in ERCC10G3). Eight guanosines were never observed for the transcripts. 

Altogether, the predominant number of guanosines in the reactions using TSOs with three riboguanines was four. In the experiments employing TSOs with three degenerate ribonucleotide positions, which better reflect the true number of G bases as the impact of the TSO guanosines is reduced, between three and four guanosines were mainly observed. Accordingly, with the template-switching conditions from the STRT protocol, we conclude that typically three to four cytidine residues were incorporated by the SSII reverse transcriptase. 

### Complexity with varying numbers of degenerate TSO positions

**Figure 3:**
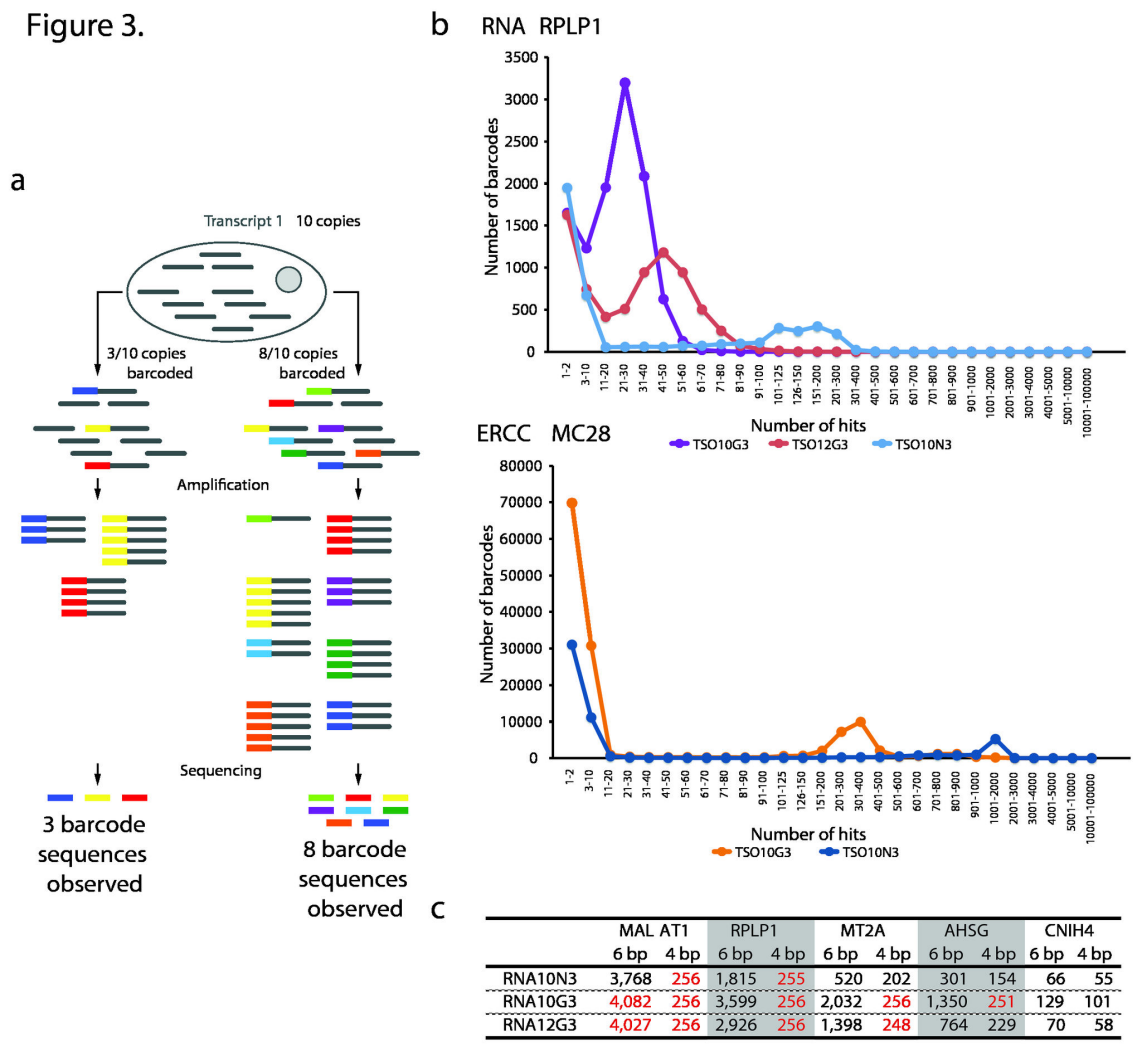
| UMI length. (a) shows the UMI principle and the output depending on reaction efficiency. If three out of the ten transcript molecules in total are labeled with the unique identifier (i.e. barcoded), only three barcodes will be observed in the sequencing data, translating into a transcript count of three for this particular transcript. Converting eight out of the ten molecules leads to the identification of eight barcodes in the sequencing reads. A UMI can become saturated if the number of transcripts copies exceeds the number of possible UMI combinations.(b) shows the distribution of barcodes for RPLP1 and spike MC28 in the performed reactions. Corresponding graphs for the other investigated transcripts are shown in Figure **S6** and Figure **S7** in File S1 for the analyzed RNA spikes. The reactions with the N10rG3 TSOs exhibited the highest complexity, followed by the reactions employing the N12rG3 and, lastly, the N10rN3 oligonucleotides. In (c) 6-base and 4-base barcodes were extracted from the MALAT1, RPLP1, MT2A, AHSG and CNIH4 reads. The red numbers indicate that the UMI has become saturated.

PCR, an integral component of the majority of genomics and transcriptomics methods, introduces bias and random errors, in particular with excessive numbers of cycles. For instance, shorter fragments are amplified to a higher extent than longer molecules and GC-neutral fragments are preferred over ones being AT- or GC-rich. One way to combat this is to use unique molecular identifiers (UMI), uniquely labeling each template molecule (Figure **3a**; [14,23-25]). UMIs can be introduced by incorporating a degenerate DNA stretch into the reaction oligonucleotides. For RNA sequencing, the UMI approach leads to each transcript molecule acquiring a unique DNA sequence in early stages of sample preparation. Counting UMIs instead of counting reads can thus eliminate biases introduced during sample preparation and PCR. The result is an improved accuracy and the possibility to measure the absolute transcript count.

UMIs can have different lengths. Two opposing factors have to be balanced. On one hand, as we observed a tendency for shorter TSOs to perform better, short UMIs may be advantageous. On the other hand, too short stretches, implying a relatively small number of possible UMI combinations and thus a low UMI complexity, can become saturated for transcripts present at higher copy numbers. Saturation occurs when number of transcript molecules exceeds the number of UMIs, leading to several transcript molecules acquiring the same UMI sequence and thus to an underestimation of the actual number of transcripts.

To study the UMI complexity and its effect for transcripts at different expression levels, we initially compared the reactions employing TSOs with degenerate stretches of 10 and 12 bases. The five transcripts outlined previously participated in this analysis. It is important to stress that this analysis is dependent on the RNA input amount, as different input amounts will, naturally, have different numbers of transcripts and thus necessitate different UMI complexity levels. In these experiments 10 ng of universal human total RNA were employed. This amount is the recommended level for the STRT technique when using purified total RNA. Consequently, a UMI complexity sufficient to cover a 10 ng total RNA experiment will certainly be adequate when the minute amounts of RNA (typically 10 - 100 pg) present in single cells are investigated. It should be noted that the number of reads analyzed was equal between the three reactions. These reads were chosen randomly from the full list of sequences. Moreover the number of reads chosen for the different transcripts was adjusted proportionally to the expression level of each gene (MALAT1 – 1M, RPLP1 – 222k, MT2A – 69k, AHSG – 18k, CNIH4 – 1,394) with the expression level being defined as the total number of reads for the transcript from all three experiments. Finally, MALAT1 was not analyzed in the RNA10N3 sample as it produced an unusually low number of reads (Table 3).

For the five analyzed transcripts, the UMI complexity was highest in the RNA10G3 reaction, followed by RNA12G3 and RNA10N3 exhibiting the lowest complexity (Figure 3B and Figure S7 in File S1). For example, for RPLP1, 9,247 UMIs were found in the RNA10G3 data set, 5,615 for RNA12G3 and 2,411 for RNA10N3 (Figure S6 in File S1). We interpreted UMIs observed less than two times as having a higher probability of being sequencing errors and removed them from the analysis. This exclusion did not, however, change the conclusions. Overall, RNA10G3 generated about twice as many distinct barcodes as RNA12G3 and RNA12G3, in turn, produced about twice as many distinct barcodes as RNA10N3. Comparing RNA10G3 and RNA12G3 the only difference was the presence of a longer UMI sequence (the total TSO length was the same). Yet, the apparent complexity of the RNA10G3 sample was greater, indicating a greater efficiency of template switching. We speculate that the longer random sequence of RNA12G3 may have generated artifacts that competed with the desired product in this reaction. The RNA10N3 reaction resulted in the lowest apparent complexity, consistent with the fact that the effective concentration of individual TSO sequences in this reaction was reduced 64-fold due to the degenerate ribonucleotide stretch. 

The two ERCC spikes MC28 and MJ-500-37 were analyzed in a similar manner and generated comparable results (Figure 3B and Figure S7 in File S1). Here, UMIs observed less than 10 times were interpreted as having a higher probability of being sequencing errors and were excluded from the analysis. As was the case with the transcripts, this exclusion did not change the conclusions. 10M randomly chosen MC28 reads and 2.9M randomly selected MJ-500-37 reads (the number of reads was expression-level adjusted) were investigated. The ERCC10G3 reaction yielded about twice as many unique barcodes as the ERCC10N3 reaction.

Next, we extracted the four and six DNA bases closest to the template-switching site (immediately preceding the ribo base region), thus mimicking a four- or six-base UMI, and counted the number of distinct UMIs found for the five different transcripts (expression level: MALAT1 > RPLP1 > MT2A > AHSG > CNIH4). The RNA10G3 reaction generated the highest number of UMIs (Figure **3c**). The four-base UMI was saturated for all analyzed genes, except for the lowest expressed CNIH4 transcript. This was evident as all, or almost all, possible 256 combinations were observed. The 6-base barcode was not enough to cover the most highly expressed MALAT1 gene as almost all 4096 combinations were detected. However, this UMI length provided sufficient complexity to cover the remaining four genes. 

The other two reactions, RNA12G3 and RNA10N3, followed a similar trend (Figure **3c**). For RNA12G3, a 4-base barcode did only cover the two least abundant transcripts (AHSG and CNIH4). On the other hand, the 6-base version gave adequate coverage for all but the MALAT1 transcript. The 6-base UMI did not become saturated for the analyzed genes in the RNA10N3 reaction. The 4-base barcode did efficiently cover MT2A, AHSG and CNIH4.

For the two analyzed ERCC spiked-in RNA species, which generated many millions of reads, the complexity afforded by neither a 4-base nor a 6-base UMI was sufficient to cover these very high expression levels (data not shown).

Consequently, a 6-base UMI is sufficient to cover all but the most highly abundant transcripts when starting with up to 10 ng of total RNA, and thus should be more than sufficient for most single-cell analyses. 

## Conclusions

Template switching is a mechanism enabling reverse transcriptases of the Moloney murine leukemia virus (MMLV) family to switch template from the RNA molecule to a secondary oligonucleotide (template-switching oligonucleotide; TSO) during first-strand cDNA synthesis. By including amplification or barcode sequences in the TSO, these features are incorporated into the final cDNA molecules. In this work, we have used our single-cell tagged reverse transcription (STRT) protocol for RNA seq, which is based on template switching, to study this mechanism. We found a strong guanosine preference at the template-switching interface, which was inversely correlated with distance from the template-switching site. As a consequence of this finding, an NGG motif for the ribo part at the 3´-end of the TSO might improve the efficiency of template switching. However, as a degenerate TSO has to be used, this enhancement may be offset by the dilution effect lowering the effective concentration of each individual oligonucleotide in the pool. Moreover, the increased TSO complexity may increase the number of mispriming events. 

Even though the guanosine preference was reduced farther from the template-switching site, four positions from this site, we still observed a slight guanosine bias. As a result, a barcode sequence near the TSO can result in bias with different reaction efficiencies for different barcodes. We also found that, as an upper limit, three to four cytidines were primarily incorporated in a nontemplate fashion. 

Unique molecular identifiers (UMIs) – random barcodes integrated in reaction oligonucleotides – uniquely labeling individual template molecules have gained marked interest recently as these can be used for absolute molecular counting to reduce PCR bias and to correct sequencing errors [14,23-25]. We found a 6-base UMI to cover all, but the most highly expressed transcripts when starting with 10 ng of total RNA. In contrast, a four-base sequence quickly became saturated. Very long UMIs are not beneficial as the larger number of sequence combinations may promote the generation of artifacts.

## Supporting Information

File S1 Figure S1, Regression analysis of TSO length data. Figure S2, Hit distributions along the analyzed transcripts. Figure S3, Composition of the ribo base portion of the TSO. Figure S4, Composition of DNA base in position 4, i.e. the DNA base adjacent to the ribo base stretch. Figure S5, Number of guanosines at the template-switching junction. Figure S6, Barcode complexity for different transcripts. Figure S7, Barcode complexity for different RNA spikes. Table S1, Primers and oligonucleotides used in this work. Table S2, p-Values for the performed optimizations. Table S3, Transcript and spike sequences used for querying the Illumina sequencing reads. Table S4, Correlation between the performed experiments. Table S5, Composition of the ribo base portion of the TSO. Table S6, Composition of DNA base in position 4, i.e. the DNA base adjacent to the ribo base stretch. Table S7, Number of guanosines at the template-switching interface.
(PDF)
