# Supplementary material for: Base Preferences in Non-Templated Nucleotide Incorporation by MMLV-Derived Reverse Transcriptases
Source: PLoS One. 2013 Dec 31;8(12):e85270. doi: 10.1371/journal.pone.0085270 (PMC3877366; doi:10.1371/journal.pone.0085270)
Supplement: File S1 — Figure S1, Regression analysis of TSO length data. Figure S2, Hit distributions along the analyzed transcripts. Figure S3, Composition of the ribo base portion of the TSO. Figure S4, Composition of DNA base in position 4, i.e. the DNA base adjacent to the ribo base stretch. Figure S5, Number of guanosines at the template-switching junction. Figure S6, Barcode complexity for different transcripts. Figure S7, Barcode complexity for different RNA spikes. Table S1, Primers and oligonucleotides used in this work. Table S2, p-Values for the performed optimizations. Table S3, Transcript and spike sequences used for querying the Illumina sequencing reads. Table S4, Correlation between the performed experiments. Table S5, Composition of the ribo base portion of the TSO. Table S6, Composition of DNA base in position 4, i.e. the DNA base adjacent to the ribo base stretch. Table S7, Number of guanosines at the template-switching interface. (PDF) [file pone.0085270.s001.pdf]

Zajac et al.

Base preferences in non-templated nucleotide incorporation by MMLV-derived reverse transcriptases

Supplementary Information

Table S1: 2
Primers and oligonucleotides used in this work.
Table S2: 3
p-Values for the performed optimizations.
Table S3: 3
Transcript and spike sequences used for querying the Illumina sequencing reads.
Table S4: 4
Correlation between the performed experiments.
Table S5: 4
Composition of the ribo base portion of the TSO.
Table S6: 5
Composition of DNA base in position 4, i.e. the DNA base adjacent to the ribobase stretch.
Table S7: 6
Number of guanosines at the template-switching interface.

Figure S1: 7
Regression analysis of TSO length data.
Figure S2: 8
Hit distributions along the analyzed transcripts.
Figure S3: 11
Composition of the ribo base portion of the TSO.
Figure S4: 12
Composition of DNA base in position 4, i.e. the DNA base adjacent to the ribobase stretch.
Figure S5: 13
Number of guanosines at the template-switching junction.
Figure S6: 14
Barcode complexity for different transcripts.
Figure S7: 15
Barcode complexity for different RNA spikes.

Table S1

Name	Sequence	Length [bp]	Ordered from
STRT Bio-T30VN	5'-Bio-aagcagtggtatcaacgcagagtCGACTTTTTTTTTTTTTTTTTTTTTTTTTTTTTTVN	58	Eurofins MWG Operon
STRT v2-7	5'-aagcagtggtatcaacgcagagtGCAGUGCU**GGACAT***rGrGrG*	40	Eurofins MWG Operon
STRT v2-7-N2	5'-aagcagtggtatcaacgcagagtUNN**GGACAT***rGrGrG*	35	Eurofins MWG Operon
STRT v2-7-N4	5'-aagcagtggtatcaacgcagagtUNNNN**GGACAT***rGrGrG*	37	Eurofins MWG Operon
STRT v2-7-N6	5'-aagcagtggtatcaacgcagagtUNNNNNN**GGACAT***rGrGrG*	39	Eurofins MWG Operon
STRT v2-7-N8	5'-aagcagtggtatcaacgcagagtUNNNNNNNN**GGACAT***rGrGrG*	41	Eurofins MWG Operon
STRT v2-7-N10	5'-aagcagtggtatcaacgcagagtUNNNNNNNNNN**GGACAT***rGrGrG*	43	Eurofins MWG Operon
STRT v2-7-N12	5'-aagcagtggtatcaacgcagagtUNNNNNNNNNNNN**GGACAT***rGrGrG*	45	Eurofins MWG Operon
STRT N10-rN3	5'-aagcagtggtatcaacgcagaguNNNNNNNNNN*rNrNrN*	36	Integrated DNA Technologies
STRT N10-rG3	5'-aagcagtggtatcaacgcagaguNNNNNNNNNN*rGrGrG*	36	Integrated DNA Technologies
STRT N12-rG3	5'-gcagtggtatcaacgcagaguNNNNNNNNNNNN*rGrGrG*	36	Integrated DNA Technologies
STRT-PCR	5'-Bio-aagcagtggtatcaacgcagagt	23	Eurofins MWG Operon

Primers and oligonucleotides used in this work.
The sequences, lengths and vendors are indicated. All sequences are written from 5'. The lowercase letters indicate the STRT amplification handle. The underlined bases for the T30 oligonucleotide denote a SalI recognition sequence. Similarly, the underlined bases for the STRT v2-7 oligonucleotide indicate a BtsI recognition site. The barcode (used in the STRT protocol to uniquely label each cell's RNA) is shown in bold and underlined typeface. Ribo bases are preceded by an 'r' and are shown in italics. An 'N' is a DNA degenerate position and an 'rN' is a RNA degenerate position. The oligonucleotides were unmodified except for the T30 oligonucleotide and STRT-PCR primer that carried a 5'-biotin.

Table S2

	Comparison	p-value
TSO amount	10 nM vs. 200 nM	1.06E-04
	40 nM vs. 200 nM	3.44E-04
	1 µM vs. 200 nM	8.77E-03
	2.5 µM vs. 200 nM	2.06E-03
	5 µM vs. 200 nM	7.09E-02
	1 µM vs. 2.5 µM	1.54E-02
	1 µM vs. 5 µM	0.973
	2.5 µM vs. 5 µM	0.133
SS enzyme	SSII vs. SSIII	1.80E-02
	SSII vs. Cycled SSIII	8.28E-03
	SSIII vs. Cycled SSIII	1.63E-02
	Cycled SSIII vs. NTC	0.861
SSII amount	200 U vs. 10 U	9.69E-03
	50 U vs. 10 U	0.470
	5 U vs. 10 U	6.95E-03
	1 U vs. 10 U	3.07E-03
	50 U vs. 200 U	1.07E-02
	5 U vs. 50 U	7.22E-03
	1 U vs. 50 U	2.95E-03

p-Values for the performed optimizations.
Student's unpaired t-test with a two-tailed distribution was used.

Table S3

	Transcipt / spike	Sequence
Transcripts	MALAT1	AGGCATTGAGGCAGCCAGCGCAGGGGCTTC
	RPLP1	CCTTTCCTCAGCTGCCGCCAAGGTGCTCGG
	MT2A	ACCACGCCTCCTCCAAGTCCCAGCGAACCC
	AHSG	CCTTTCCCAGCAGAGCACCTGGGTTGGTCC
	CNIH4	AGGAGCGGCGGCGACGGAGGAGGAGGATGG
Spi-kes	MC28	GGAATTCTCCAGATTACTTCCATTTCCGCC
	MJ-500-37	GGAATTCTGGACATTAATTAGGGCTGAAAG

Transcript and spike sequences used for querying the Illumina sequencing reads.
The human transcript sequences were obtained from definitions in the refFlat.txt file for hg19 from the UCSC Genome Browser. The spike sequences were provided by Life / Ambion. Apart from AHSG that starts with base number three in the transcript defined by refFlat.txt for hg19 and MALAT1 where the main template-switching peak is 1300 bp from the 5'-end, the other query sequences coincide with the defined 5'-end of the transcripts.

Table S4

Reaction	RNA10G3	RNA12G3	RNA10N3
RNA10G3	**1.000**	0.999	0.999 (0.484)
RNA12G3		**1.000**	1.000 (0.506)
RNA10N3			**1.000**

Correlation between the performed experiments.
R2 values are shown. These correlations are based on the five investigated transcripts: MALAT1, RPLP1, MT2A, AHSG and CNIH4. For the correlations involving RNA10N3, the MALAT1 gene was omitted as it generated an unusually low number of reads in this reaction. The R^2 values featuring MALAT1 are shown in parentheses.

Table S5

	Guanosine percentage [%]			Percentage [%]				
**Spike / Transcript**	**Position 1**	**Position 2**	**Position 3**	**AGG**	**CGG**	**GGG**	**TGG**	**NGG**
MC28	98	80	46	18	11	38	11	79
MJ-500-37	97	79	54	16	10	43	9	77
MALAT1	91	85	58	15	11	44	8	77
RPLP1	93	84	62	11	9	52	7	79
MT2A	91	87	63	10	12	53	4	79
Average	*94*	*83*	*57*	*14*	*11*	*46*	*8*	*78*

Composition of the ribo base portion of the TSO.

Table S6

	Position 4 percentage [%]					
	Spike / Transcript	A	C	G	T	Order
ERCC10N3	MC28	20	27	31	22	G > C > T > A
	MJ-500-37	18	25	35	22	
ERCC10G3	MC28	25	19	33	23	G > A > T > C
	MJ-500-37	24	20	35	22	
RNA10N3	MALAT1	16	33	32	19	G > C > T > A
	RPLP1	15	31	35	20	
	MT2A	14	35	38	14	
RNA10G3	MALAT1	21	22	36	22	G > C/T/A
	RPLP1	20	20	38	23	
	MT2A	19	20	40	21	
RNA12G3	MALAT1	15	22	38	25	G > T > C > A
	RPLP1	16	20	40	25	
	MT2A	12	22	40	26	

Composition of DNA base in position 4, i.e. the DNA base adjacent to the ribobase stretch.

Table S7

Number of guanosines	Percentage [%]									
	ERCC10N3		ERCC10G3		RNA10N3		RNA10G3		RNA12G3	
	MC28	MJ-500-37	MC28	MJ-500-37	RPLP1	MT2A	RPLP1	MT2A	RPLP1	MT2A
2	18.86	20.31	0.65	0.76	20.14	22.56	0.48	0.31	0.51	0.39
3	*35.98*	*32.33*	4.29	6.62	*27.96*	*27.57*	16.71	23.01	16.99	26.12
4	*28.18*	*28.54*	*62.88*	*59.93*	*32.95*	*32.22*	*56.85*	*58.40*	*57.73*	*57.67*
5	14.37	16.69	28.08	28.71	17.72	17.65	24.49	18.16	23.61	15.35
6	2.23	1.71	3.53	3.48	1.25	0.00	1.47	0.02	1.09	0.47
7	0.35	0.30	0.52	0.43	0.00	0.00	0.00	0.11	0.07	0.01
8	0.04	0.12	0.05	0.07	0.00	0.00	0.00	0.00	0.00	0.00

Number of guanosines at the template-switching interface.

Figure S1

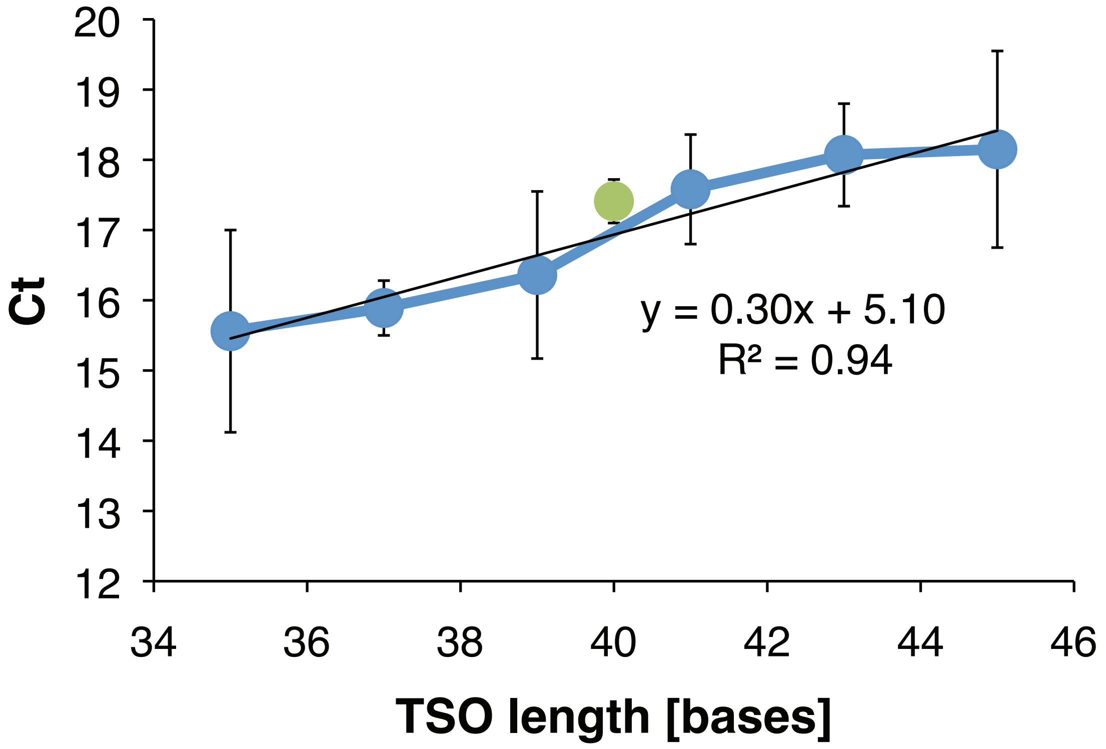

Regression analysis of TSO length data.

Figure S2

MALAT1

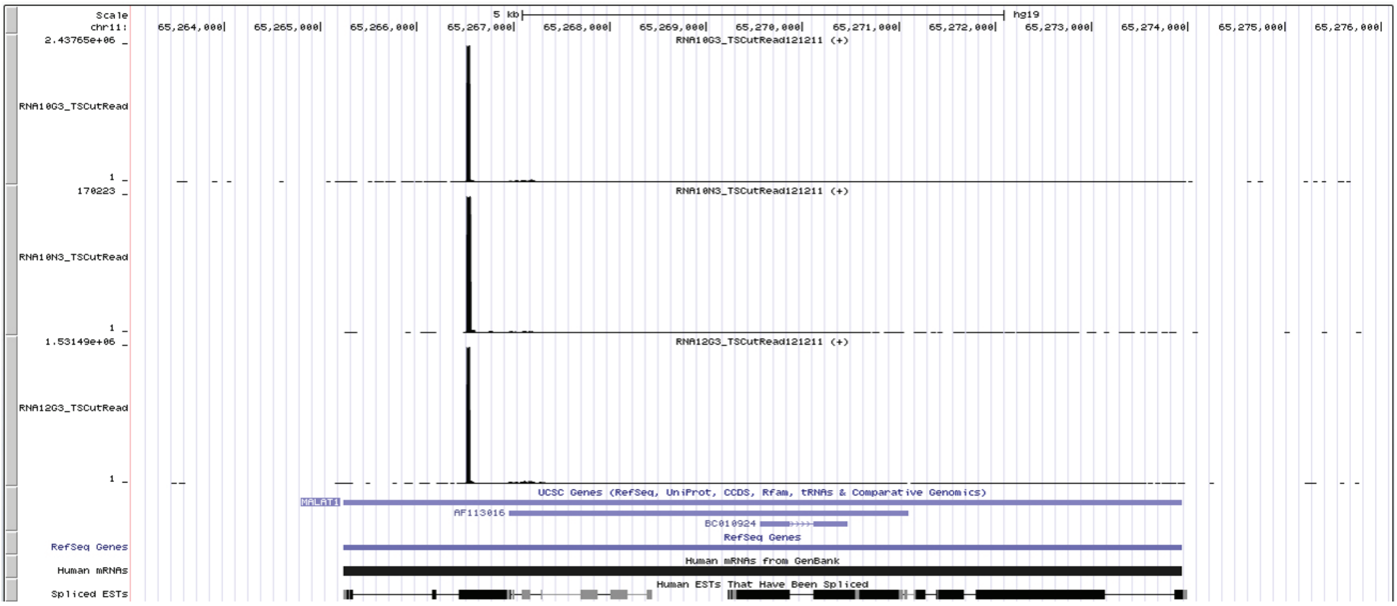

RPLP1

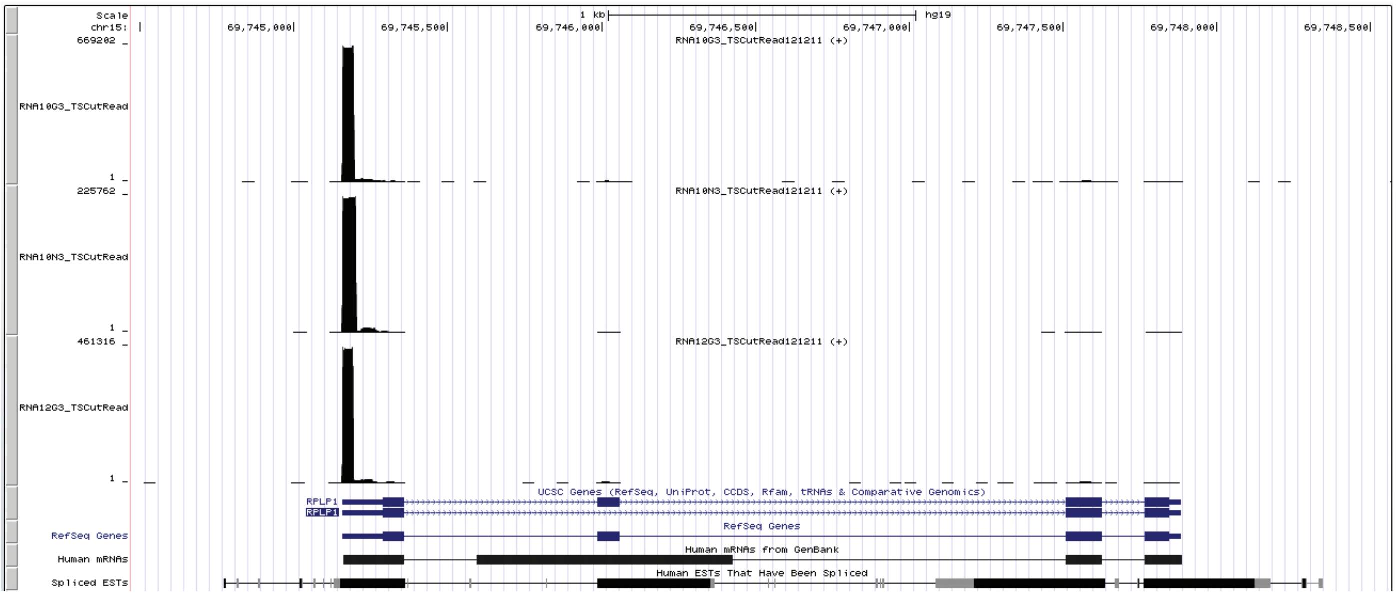

Hit distributions along the analyzed transcripts.
Images from Genome Browser are shown. The upper tracks correspond to the RNA10G3 reaction, the middle tracks to the RNA10N3 reaction and the bottom tracks to the RNA12G3 reaction. The hit distributions are highly similar.

Figure S2 (continued)

MT2A

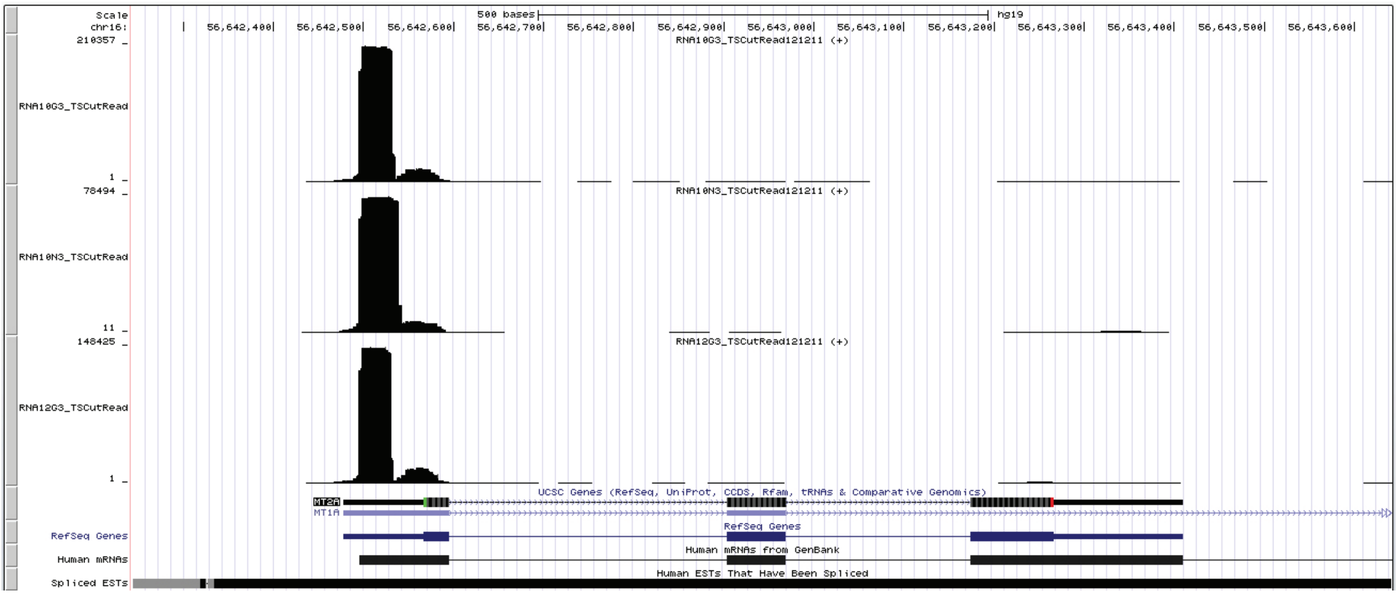

AHSG

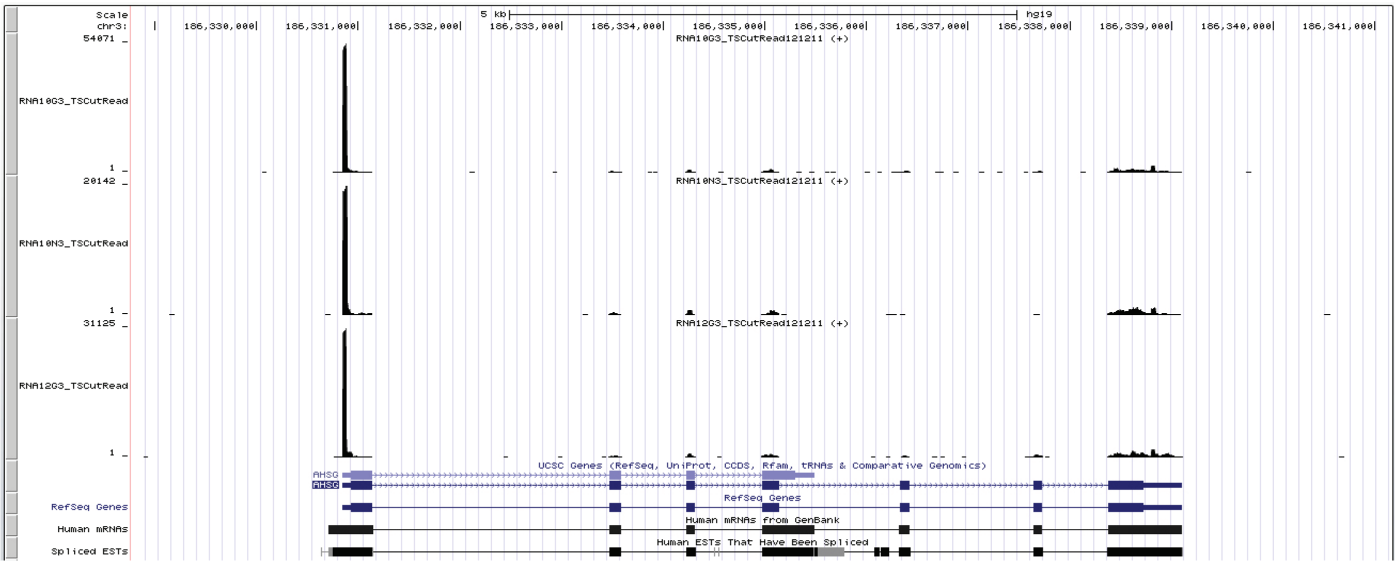

Figure S2 (continued)

CNIH4

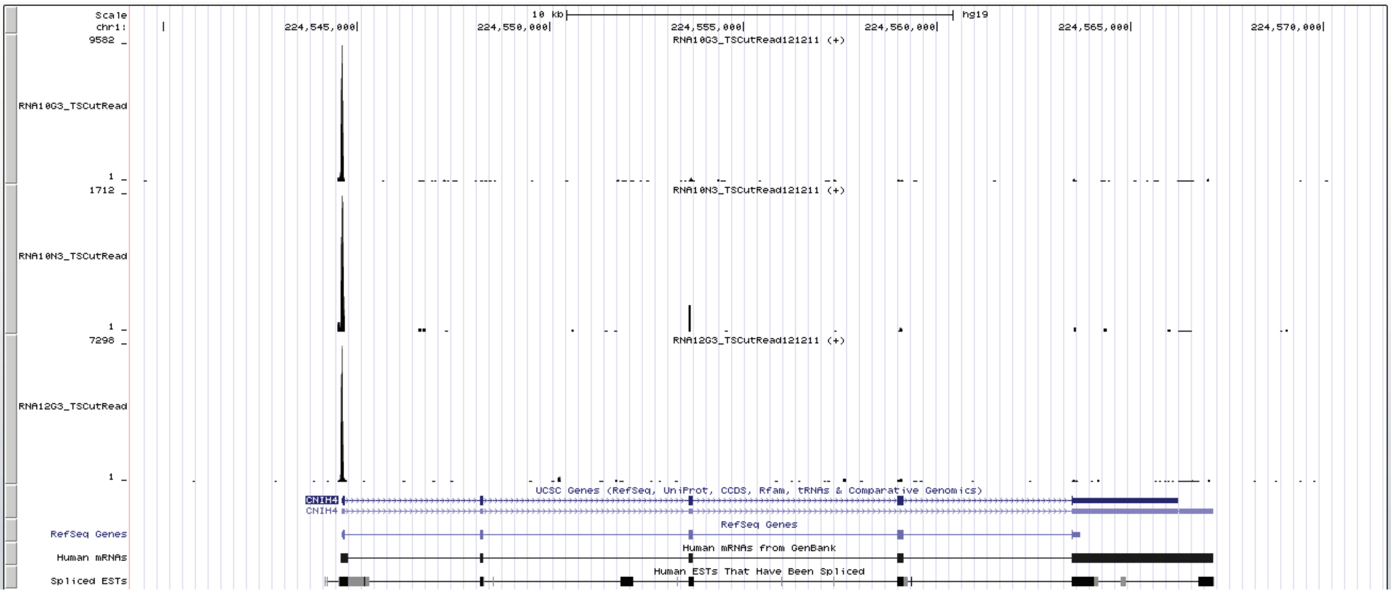

Figure S3

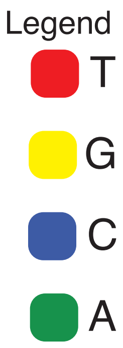

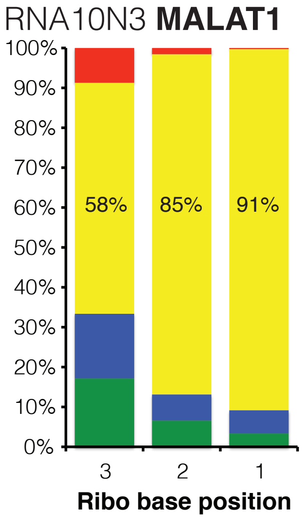

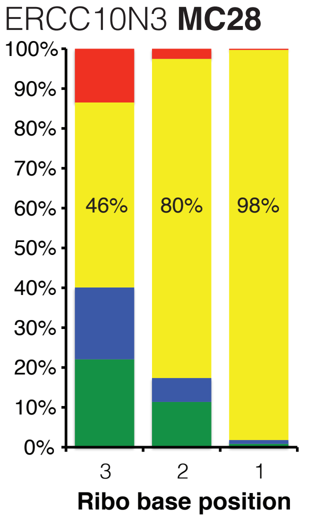

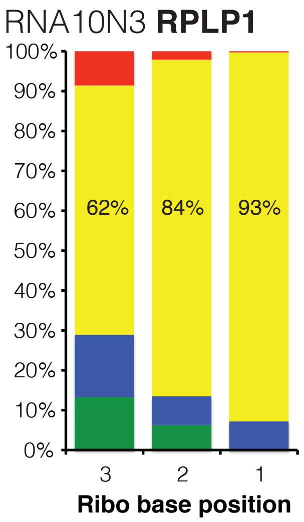

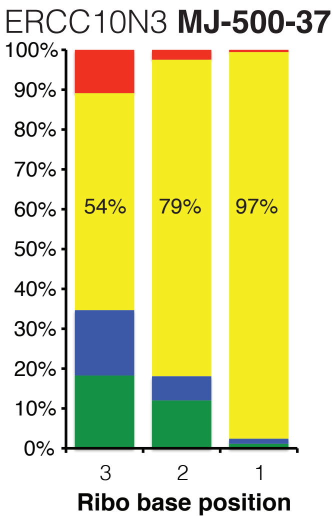

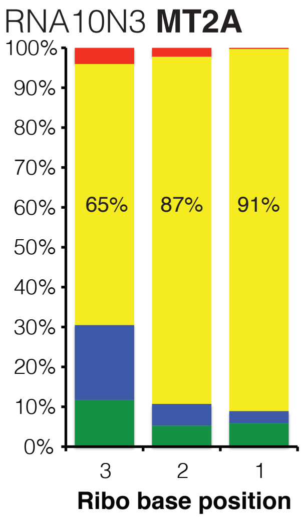

Composition of the ribo base portion of the TSO.
Position 1 represents the ribo base at the template-switching junction. In all cases, G is the preferred nucleotide in the three ribo positions of the TSO. The preference for G decreases as the distance from the template-switching site increases.

Figure S4

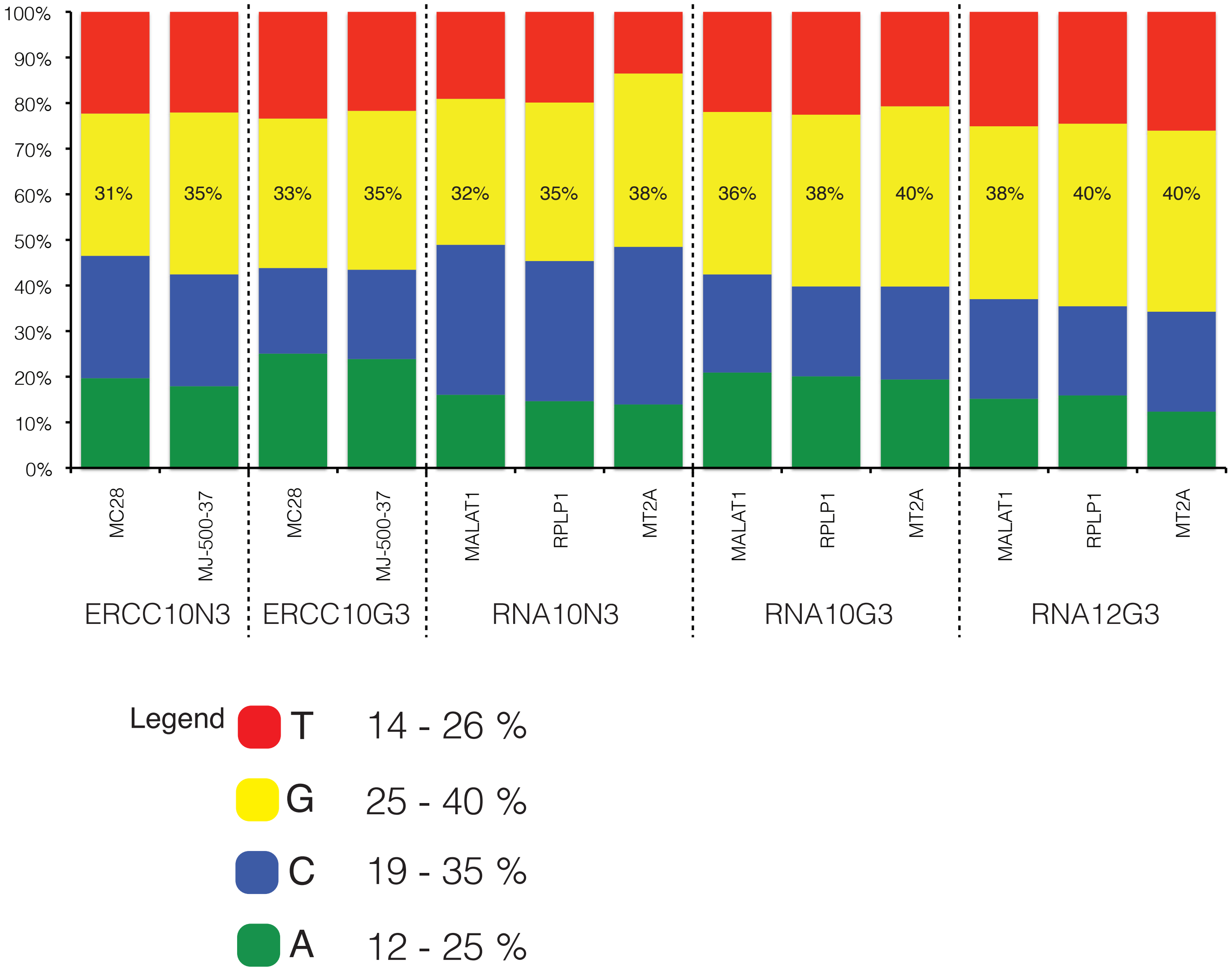

Composition of DNA base in position 4, i.e. the DNA base adjacent to the ribo base stretch.
Position 4 is the first DNA position in the TSO as seen from the template-switching site. As such, it is located adjacent to the ribo base portion. G is the preferred nucleotide in this position.

Figure S5

RPLP1

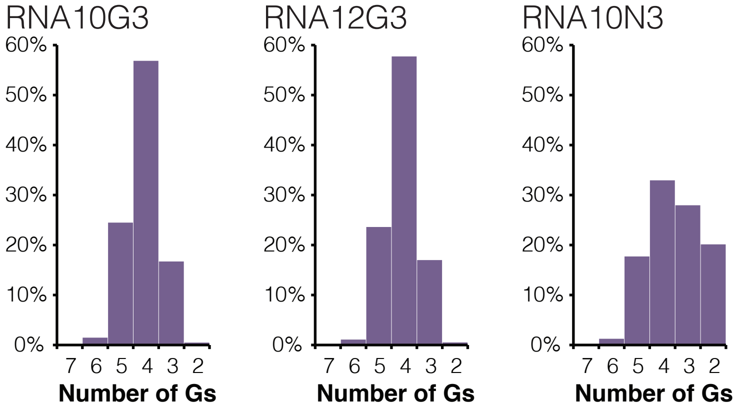

MT2A

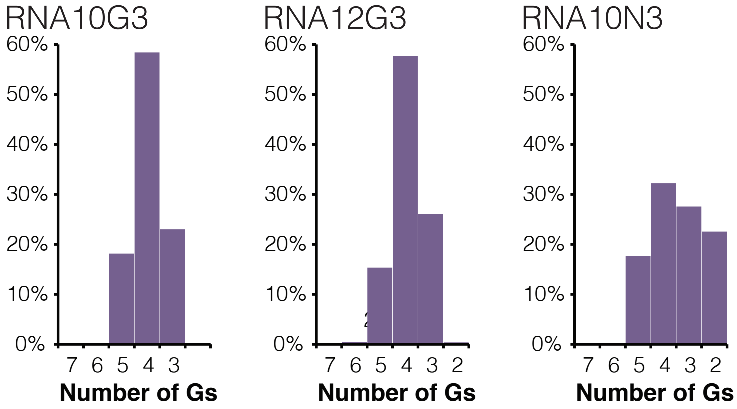

MC28

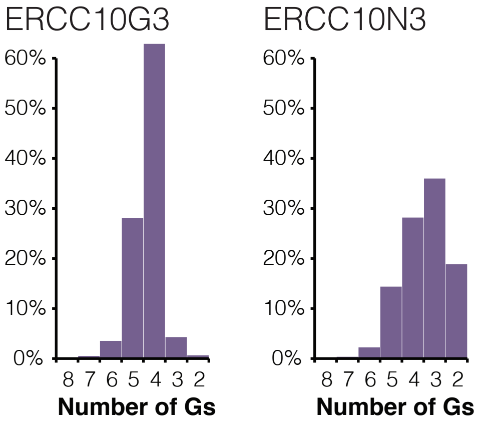

MJ-500-37

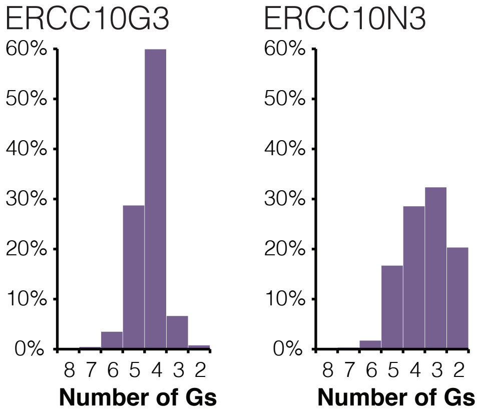

Number of guanosines at the template-switching junction. The graphs show the distribution of the number of guanosines observed in the sequencing data for the analyzed transcripts and RNA spike molecules. In general, three to four Gs are observed most frequently.

Figure S6

MALAT1

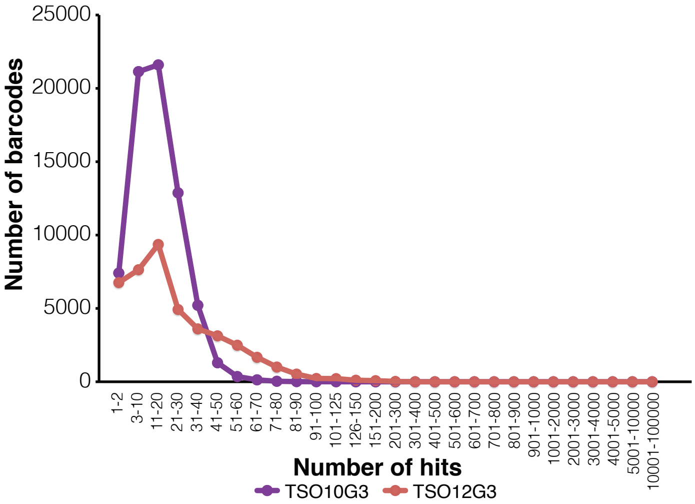

AHSG

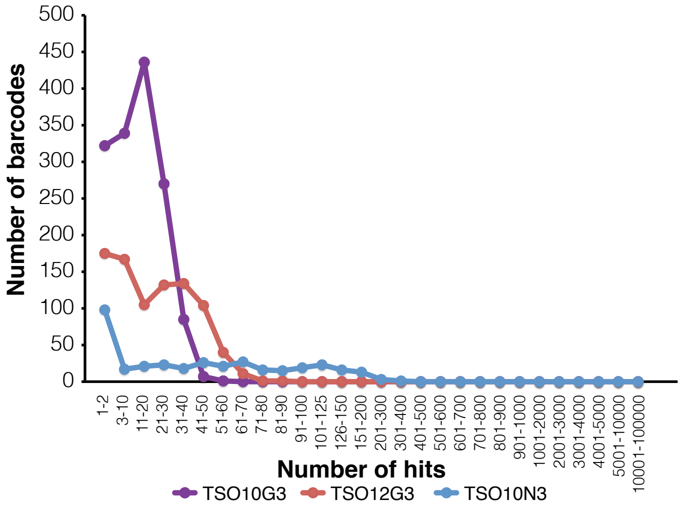

RPLP1

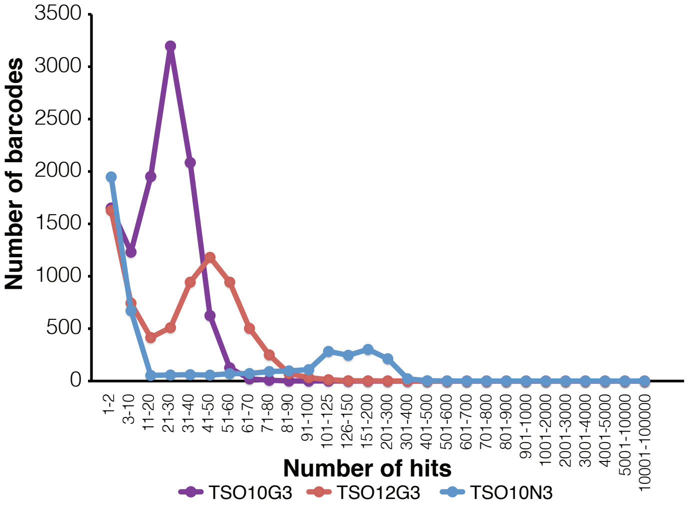

CNIH4

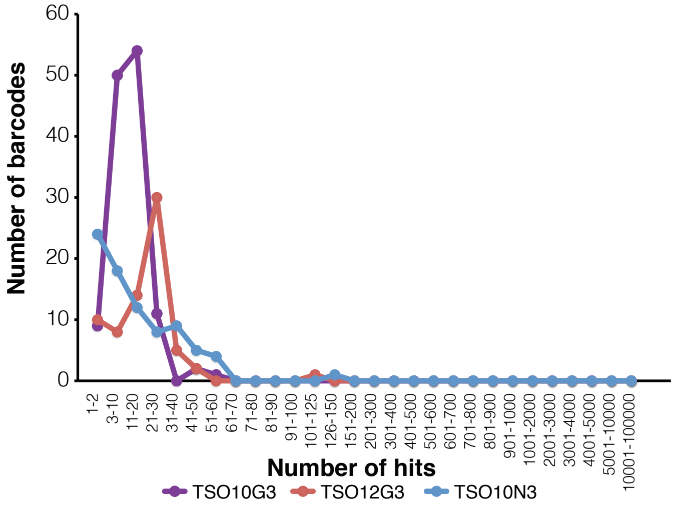

MT2A

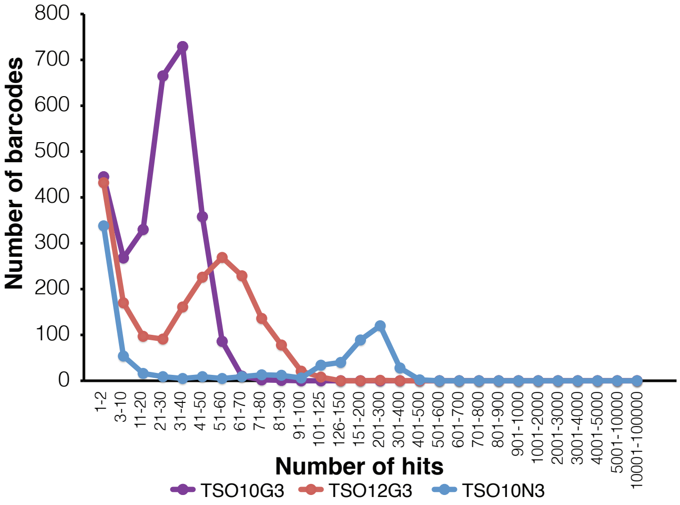

Barcode complexity for different transcripts.
The graphs show the barcode distribution in the three reactions employing total RNA for the analyzed transcripts. The reaction with TSO10G3 exhibits the highest apparent complexity, followed by the reaction with TSO12G3 and with the TSO10N3 reaction showing the lowest apparent complexity.

Figure S7

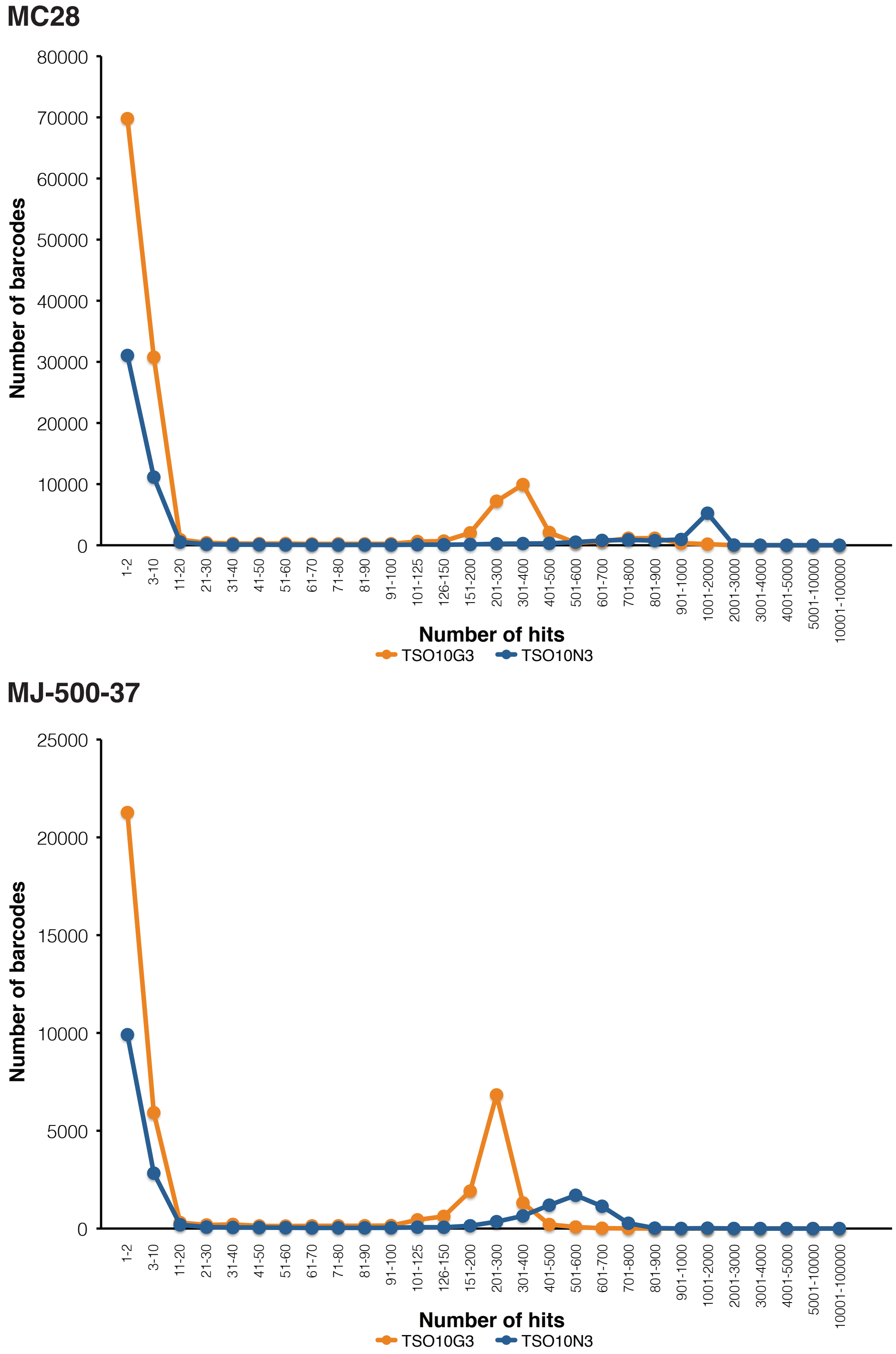

Barcode complexity for different RNA spikes.
The graphs show the barcode distribution in the two reactions employing ERCC spikes for the analyzed spike molecules. The reaction with TSO10G3 exhibits higher apparent complexity than the reaction with TSO12G3.
